# Error and Error Mitigation in Low-Coverage Genome Assemblies

**DOI:** 10.1371/journal.pone.0017034

**Published:** 2011-02-14

**Authors:** Melissa J. Hubisz, Michael F. Lin, Manolis Kellis, Adam Siepel

**Affiliations:** 1 Department of Biological Statistics and Computational Biology, Cornell University, Ithaca, New York, United States of America; 2 Broad Institute of Massachusetts Institute of Technology and Harvard University, Cambridge, Massachusetts, United States of America; 3 Computer Science and Artificial Intelligence Laboratory, Massachusetts Institute of Technology, Cambridge, Massachusetts, United States of America; 4 Cornell Center for Comparative and Population Genomics, Cornell University, Ithaca, New York, United States of America; Aarhus University, Denmark

## Abstract

The recent release of twenty-two new genome sequences has dramatically increased the data available for mammalian comparative genomics, but twenty of these new sequences are currently limited to ∼2× coverage. Here we examine the extent of sequencing error in these 2× assemblies, and its potential impact in downstream analyses. By comparing 2× assemblies with high-quality sequences from the ENCODE regions, we estimate the rate of sequencing error to be 1–4 errors per kilobase. While this error rate is fairly modest, sequencing error can still have surprising effects. For example, an apparent lineage-specific insertion in a coding region is more likely to reflect sequencing error than a true biological event, and the length distribution of coding indels is strongly distorted by error. We find that most errors are contributed by a small fraction of bases with low quality scores, in particular, by the ends of reads in regions of single-read coverage in the assembly. We explore several approaches for automatic sequencing error mitigation (SEM), making use of the localized nature of sequencing error, the fact that it is well predicted by quality scores, and information about errors that comes from comparisons across species. Our automatic methods for error mitigation cannot replace the need for additional sequencing, but they do allow substantial fractions of errors to be masked or eliminated at the cost of modest amounts of over-correction, and they can reduce the impact of error in downstream phylogenomic analyses. Our error-mitigated alignments are available for download.

## Introduction

The field of comparative mammalian genomics has been given an enormous boost by the recent release of genome assemblies for 22 previously unsequenced species of eutherian (placental) mammals (2× Mammals Consortium, in prep.). These new assemblies increase the number of sequenced eutherian species by nearly fourfold, and provide an opportunity for many new functional and evolutionary insights in mammalian genomics. Nevertheless, twenty of these twenty-two genome sequences are currently available only as low- coverage (∼2×) assemblies (Table 1), produced using traditional, capillary sequencing methods. Most of these twenty will eventually be sequenced to higher coverage (see http://www.genome.gov/10002154), but even with advances in next-generation sequencing, the task of “topping them up” remains daunting and will require years to complete. In the meantime, the data available for mammalian comparative genomics will be heavily dominated by low-coverage sequence.

**Table 1:** Species and genome assemblies considered in this study.

Species^a^	Assembly^b^	Coverage^c^	ENCODE^d^
human	hg18	complete	–
chimp	panTro2	6.0×	–
rhesus	rheMac2	5.1×	–
tarsier	tarSyr1	2.0×	–
mouse lemur	micMur1	1.9×	43
bushbaby	otoGar1	1.9×	44
tree shrew	tupBel1	1.9×	4
mouse	mm9	complete^e^	–
rat	rn4	complete^e^	–
kangaroo rat	dipOrd1	2.0×	–
guinea pig	cavPor3	6.8×	41
squirrel	speTri1	1.9×	44
rabbit	oryCun1	2.0×	43
pika	ochPri2	1.9×	–
alpaca	vicPac1	2.0×	–
dolphin	turTru1	2.0×	–
cow	bosTau4	7.1×	–
horse	equCab2	6.8×	–
cat	felCat3	1.9×	43
dog	canFam2	7.6×	–
microbat	myoLuc1	1.8×	41
megabat	pteVam1	2.6×	5
hedgehog	eriEur1	1.9×	39
shrew	sorAra1	1.9×	41
elephant	loxAfr2	1.9×	44
rock hyrax	proCap1	2.2×	6
tenrec	echTel1	1.9×	42
armadillo	dasNov2	2.0×	44
sloth	choHof1	2.0×	–
opossum	monDom4	6.8×	–
chicken	galGal3	6.6×	–
tetraodon	tetNig1	7.9×	–

a Abbreviated common names used by the 2× Mammals Consortium are listed here. In some cases, different names have been used in the ENCODE project (see Table S1).

b UCSC designation.

c Reported average coverage.

d Number of ENCODE regions for which high-quality sequence is available, or“–” if ENCODE sequence is unavailable.

e The mouse and rat assemblies are considered “essentially complete” but some improvements are still underway (see http://www.ncbi.nlm.nih.gov/genome/assembly/grc/mouse/ and http://www.hgsc.bcm.tmc.edu/project-species-m-Rat.hgsc?pageLocation=Rat).

While these low-coverage assemblies are valuable for many purposes, reduced sequencing redundancy has some inevitable costs. For example, low-coverage assemblies have decreased levels of contiguity, which can severely limit their usefulness in identifying rearrangements, duplications, and repetitive elements [1]. In addition, low-coverage assemblies necessarily have elevated levels of *sequencing error*—that is, miscalled bases and erroneous insertions and deletions, which might otherwise be corrected through redundant sequencing of the same genomic region. This issue of sequencing error in 2× genomes is our focus in this article. To our knowledge, this issue has not been studied in detail, despite its potential importance in many applications in comparative genomics, including phylogenetic modeling, the detection of positive selection, and comparative gene finding. Several potential limitations of low-coverage assemblies were examined by Margulies et al. [2], but their study focused on assembly and alignment error, and its influence on the detection power for conserved elements in mammalian genomes.

Considered more broadly, sequencing error is, of course, not a new concern. Early sequencing studies tended to consider the issue of error informally and qualitatively [3], but by the late 1980s and early 1990s, the need for a more rigorous, quantitative treatment became apparent. At this time, efforts were undertaken to measure the overall error rates in the growing sequence databases [4], to detect errors automatically in protein-coding sequences [5], and to incorporate explicit models of sequencing error into algorithms for alignment and assembly [6], [7]. By the late 1990s, *phred* quality scores [8] (see also [9]) had become established as a standard, highly accurate method for quantifying the probability of error at each nucleotide in a sequence. A trend toward high-coverage sequencing led to reduced concern with sequencing error in the early 2000s, and error was essentially ignored in most of the comparative genomic studies of mammals carried out during this period [10]–[12]. More recently, however, error has re-emerged as an important concern in sequence analysis, in part because errors occur at elevated rates in the reads produced by many next-generation sequencing technologies. Error is an important consideration in applications in population genetics, in which care is required to distinguish errors from polymorphisms [13]–[16], in mapping short read sequences to reference genomes [17], in *de novo* assembly from short reads [18], and in improving genome finishing [19]. In addition, sequencing error has been shown to have important effects in comparative genomics, particularly in genomic scans for positive selection [20], [21]. One recent study has shown that indel error occurs at appreciable rates in the draft-quality orangutan and chimpanzee genomes assemblies [22].

In this article, we estimate the rates at which substitution, insertion, and deletion errors occur in the current 2× assemblies, by comparing them with high-coverage sequences from the ENCODE project [23]. We show that these assemblies exhibit modest but non-negligible levels of sequencing error, which, if ignored, can produce biases in downstream phylogenomic analyses. We show that the error present in these assemblies is strongly concentrated in regions of single-read coverage, particularly at the ends of reads. Next, we explore the possibility of applying automatic methods for *sequencing error mitigation* (SEM), including masking of bases likely to be miscalled and “correction” of indels likely to be spurious. We show that even quite simple strategies are capable of eliminating fairly large fractions of sequencing errors at the cost of a modest amount of “over-correction”. At least for some downstream analyses, the benefits of eliminating true errors appear to outweigh the costs of obscuring a fraction of true genomic mutations. Our error-mitigated alignments of 32 vertebrate genomes are available from http://compgen.bscb.cornell.edu/projects/32way-masked/, and our software for SEM is available by request.

## Results

### Assessment of sequencing error

We measured sequencing error in the newly available low-coverage (∼2×) genome by comparing them with corresponding “comparative-grade” sequences from the ENCODE project [23], [24] (Table 1). The bacterial artificial chromosome (BAC)-based ENCODE sequences exhibit high quality, with a sequencing error rate of ∼1 in 10,000 bases [25], and high coverage, with representation from 31 mammalian species (see http://www.nisc.nih.gov/projects/encode/) and 44 genomic regions spanning ∼30 Mbp (∼1%) of the human genome. We restricted our comparison to the fourteen 2× species that have been sequenced in the ENCODE regions, additionally including the ∼7× guinea pig for comparison (Table 1 and Table S1). To our knowledge, there is no comparable “gold standard” available for the remaining six 2× species.

For each of the fifteen species of interest, we aligned the entire 2× assembly against the ENCODE sequences for that species, then applied a series of filters to ensure that corresponding regions were aligned with high confidence (see Materials and Methods). These alignments could then be examined for mismatching bases and alignment gaps, with mismatches indicating potential miscalled bases, gaps in the ENCODE sequences indicating potential insertion errors, and gaps in the 2× assemblies indicating potential deletion errors. After filtering, the pairwise alignments for the 2× species contained 2.1–20.1 Mbp of aligned sequence per species, depending in large part on the ENCODE sequencing coverage (Table S2). In most cases, half or more of the bases in the available ENCODE sequences were in alignment with the 2× assemblies (fractions ranged from 38–84%), indicating good coverage in the 2× assemblies and a reasonably sensitive alignment procedure, despite the use of conservative filters. These fractions are probably influenced by various factors, including the degree of contiguity of both the ENCODE and 2× genomes (which will affect alignability), the repetitive content of the genomes, and differences in the DNA sources for the two sequencing projects (see below).

#### Error rates

**Figure 1:**
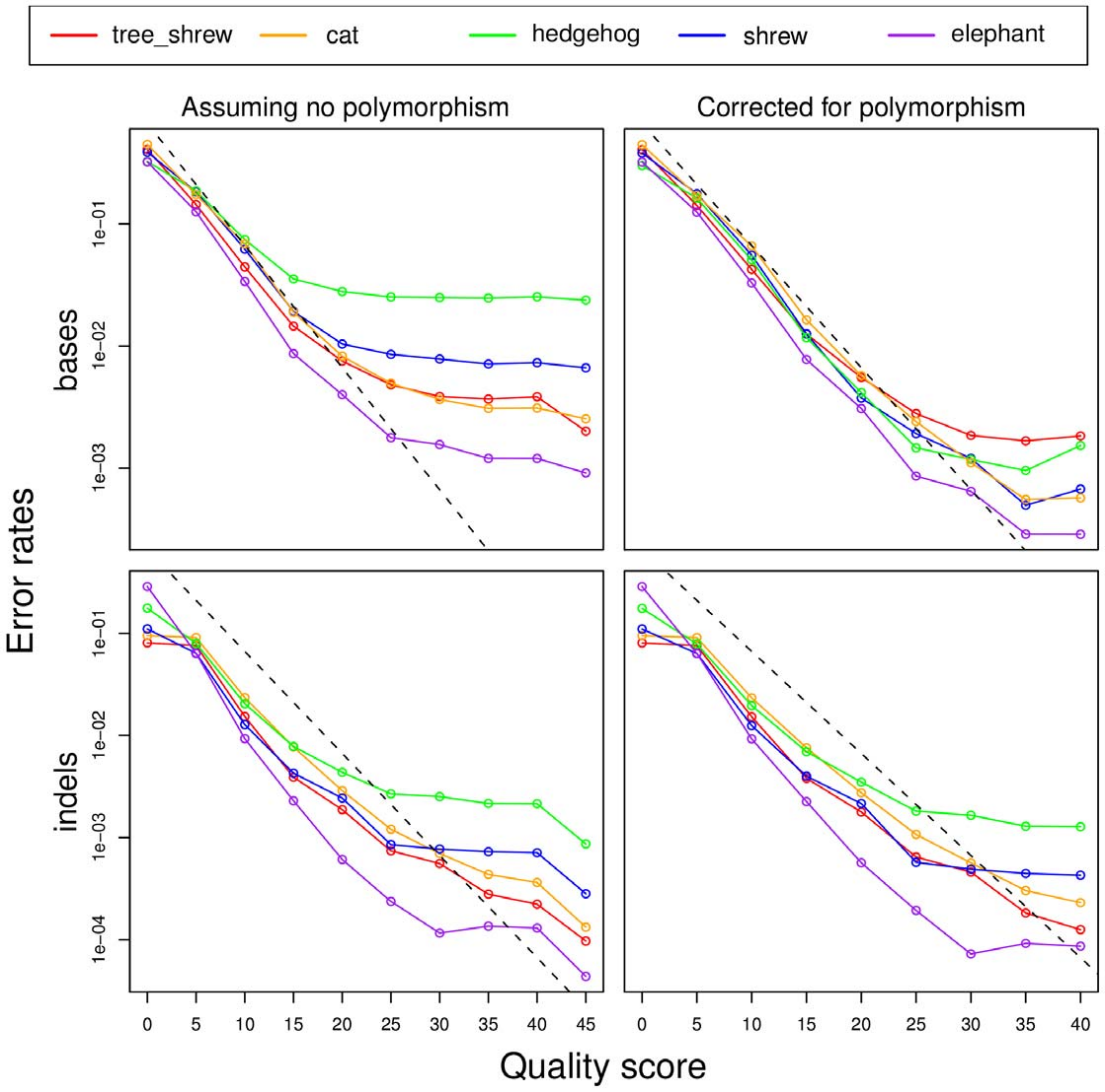
Quality scores vs. error rates in selected 2× genomes. Error rates were estimated from alignments with high-quality ENCODE sequences. Separate plots are shown for basecall (top) and indel (bottom) errors, before (left) and after (right) correcting for polymorphisms (see text). The quality score of an insertion is taken to be the minimum score of the inserted bases and the 10 nearest neighboring bases (5 on each side), while the score of a deletion is the minimum score of just the 10 neighboring bases. The dashed line shows the rates implied by nominal quality scores (i.e., error rates of 10^−*q*/10^ for each score *q*). Note that these predicted rates include both base-call and indel errors [8], so they are expected to be slightly larger than the true rates for the individual error types. The excess of errors at large quality scores could result from an underestimation of polymorphism (perhaps because the high quality regions used to estimate polymorphism are depleted for segregating sites), or from an overestimation of quality scores (perhaps because the assembler assumes independence of reads in estimating aggregate scores). The numbers of events were sufficiently large that sampling errors were small relative to the dependency on quality scores.

The mismatch rates for aligned 2× and ENCODE bases ranged from 2.6 (elephant) to 25.1 (hedgehog) mismatches per kb (Table 2), while the indel rates showed somewhat less variation, with insertion rates from 0.22 (megabat) to 0.86 (hedgehog) per kb and deletion rates from 0.24 (tenrec) to 0.77 (microbat) per kb. Both mismatch and indel rates generally decreased with increasing quality scores (Figure 1), as expected, but the limiting rates—at the highest quality scores—differed considerably among species. This is because the observed differences not only reflect sequencing errors, which are expected to occur at similar rates across species, but they also reflect true genomic differences between the individuals sequenced for the 2× and ENCODE projects, which will depend on the diversity of the sampled populations, the relatedness of the sampled individuals, and other factors. Indeed, the largest mismatch rates at high quality scores were observed with the hedgehog, for which separate (but closely related) species were sequenced in the ENCODE (middle-African hedgehog) and 2× (European hedgehog) projects. The second largest mismatch rates were observed with the microbat, for which some evidence of elevated levels of intraspecific genetic variation has been reported [26], while the smallest mismatch rates occurred with the African savannah elephant, which has been reported to have low average genetic diversity [27].

**Table 2:** Error rates per kilobase, raw and corrected.

Species	Cov.^a^	Basecalls			Insertions			Deletions		
		Raw^b^	Poly.^c^	Corr.^d^	Raw^b^	Poly.^c^	Corr.^d^	Raw^b^	Poly.^c^	Corr.^d^
armadillo	2.0×	3.06	1.48	1.58	0.40	0.03	0.37	0.33	0.03	0.30
tenrec	1.9×	3.19	1.08	2.11	0.47	0.02	0.45	0.24	0.02	0.22
hedgehog	1.9×	25.10	23.71	1.39	0.86	0.43	0.43	0.76	0.44	0.32
cat	1.9×	3.72	2.53	1.19	0.33	0.06	0.27	0.28	0.07	0.21
elephant	1.9×	2.61	0.91	1.70	0.41	0.02	0.39	0.42	0.02	0.40
mouse lemur	1.9×	3.00	1.92	1.08	0.26	0.03	0.23	0.46	0.05	0.41
microbat	1.8×	15.04	11.61	3.43	0.57	0.16	0.41	0.77	0.30	0.47
rabbit	2.0×	5.63	4.08	1.55	0.44	0.06	0.38	0.30	0.09	0.21
bushbaby	1.9×	4.94	2.59	2.35	0.43	0.04	0.39	0.75	0.05	0.70
rock hyrax	2.2×	3.72	2.68	1.04	0.25	0.04	0.21	0.42	0.06	0.36
megabat	2.6×	3.73	3.01	0.72	0.22	0.06	0.16	0.37	0.08	0.29
shrew	1.9×	8.04	6.62	1.42	0.35	0.13	0.22	0.39	0.15	0.24
squirrel	1.9×	5.64	3.16	2.48	0.51	0.05	0.46	0.63	0.08	0.55
tree shrew	1.9×	4.43	2.01	2.42	0.37	0.04	0.33	0.56	0.06	0.50
guinea pig^e^	6.8×	0.35	0.14	0.21	0.04	0.00	0.04	0.04	0.00	0.04

a Reported average coverage of 2× assembly.

b Based on observed differences between aligned 2× and ENCODE sequences. Note that, because estimates are based on >2Mb of aligned sequence in all cases and >10Mb in most cases (Table S2), the contribution of sampling error to the estimates shown here is negligible (standard errors are typically ≈0.01 per kb).

c Estimate of polymorphism rate (based on highest quality bases).

d Corrected error rate, equal to raw difference rate minus estimated polymorphism rate.

e The guinea pig is shown for comparison. The genome assembly reflects −7× coverage. The low estimated polymorphism rate may result from the use of the same inbred line in the 2× and ENCODE projects (unconfirmed).

To disentangle the contributions of polymorphism and sequencing error, we used the observed difference rates at the highest quality bases (quality score ≥45) as rough estimates of polymorphism rates, under the assumption that error is negligible compared with polymorphism at these bases. We then subtracted these estimates from the overall observed rates to obtain approximate polymorphism-corrected error rates. As a side-benefit, this approach should at least partly correct for ENCODE sequencing errors and 2×/ENCODE alignment errors, as long as these occur at similar rates in high quality and low quality regions of the 2× assemblies. This method predicts nucleotide diversity levels ranging from ∼1–7×10^−3^ for most species (with somewhat larger values of 1.2 and 2.4×10^−2^ for microbat and hedgehog, respectively; Table 2), in reasonable agreement with an average diversity of π≈5×10^−3^ for nuclear DNA in mammals [28]. After this adjustment, the estimated base-call error rates are much more concordant across species, at 0.72–3.43 per kb, and the indel error rates are slightly more concordant, at 0.16–0.46 per kb (insertions) and 0.21–0.70 per kb (deletions). The 2× species with slightly higher coverage, such as megabat (2.6×) and rock hyrax (2.2×), have the lowest error rates, as expected. In addition, both types of error rates are in substantially better agreement with the nominal quality scores (Figure 1). The polymorphism-corrected error rates are used throughout the remainder of this paper, unless otherwise noted.

**Figure 2:**
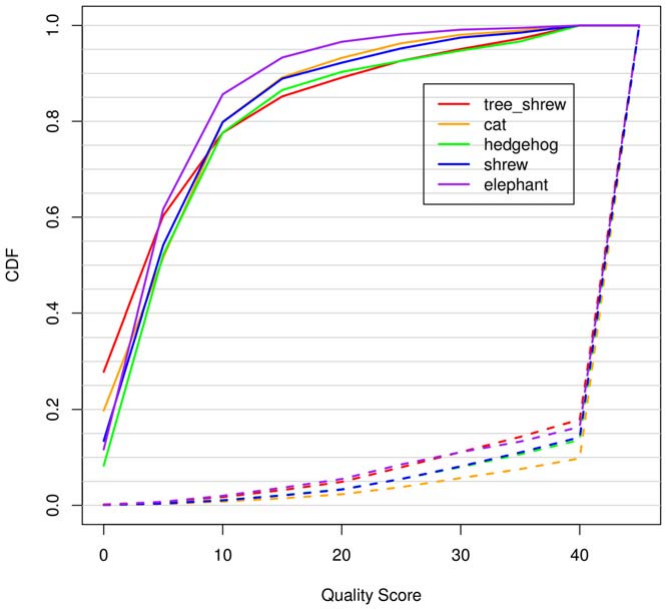
Cumulative distribution functions (CDFs) of quality scores and basecall errors. Results are shown for four representative 2× assemblies. At each quality score threshold *q*, shown on the *x* axis, the dashed lines indicate the fraction of all bases having quality score *q*, and the solid lines indicate the fraction of all basecall errors that are associated with bases having quality score *q*. This graph highlights the fact that most bases have high quality scores, but most errors derive from bases with low quality scores.

Even with only ∼2× average genomic coverage, these assemblies predominantly consist of high-quality bases. Roughly 82% of bases, across all of the assemblies, have quality scores ≥45 (the highest category considered in our analysis; see Materials and Methods), and only ∼4% have scores <20 (Figure 2). Nevertheless, these low-quality bases are sufficiently error-prone that they make a highly disproportionate contribution to the overall error rates. Indeed, once polymorphisms are corrected for, 88.4% of basecall errors occur in bases with quality scores <20. The situation with indels is similar, with 75.2% of erroneous insertions and 73.9% of erroneous deletions being accounted for by the small fraction of bases having scores <20.

### Model for coverage and error

**Figure 3:**
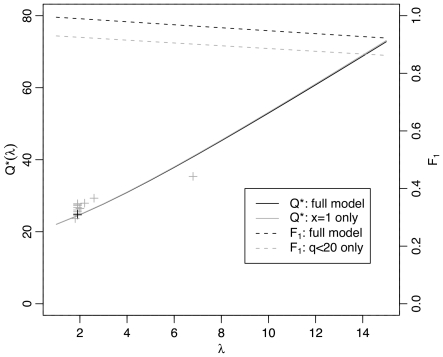
Quality as a function of coverage, according to theoretical model. Shown are expected coverage (*λ*) versus expected sequence quality (Q^*^(*λ*); solid curves, labels on left) and expected fraction of error contributed by single-coverage regions of an assembly (*F*_1_; dashed curves, labels on right). Sequence quality is expressed in *phred* units, as Q*(*λ*) = 10 log_10_ err(*λ*), where err(*λ*) is the error probability per base. The solid black curve shows that expected sequence quality increases nearly linearly with expected coverage, at a rate of ∼3.62 *phred* units per additional 1× of coverage, corresponding to a 10−3.62/lO≈43% decrease in the error rate. The nearly identical solid gray curve considers only errors from single-coverage (*x* = 1) portions of the assembly, demonstrating that average error rates are almost completely determined by single-coverage regions of the assembly, and increases in quality are almost completely attributable to decreases in single-read coverage. The dashed black curve shows the fraction of all errors that come from single-coverage bases (F_1_), and the dashed gray curve shows the fraction that come from low quality (*q*<20) single-coverage bases, which occur predominantly at the ends of reads. Notice that, even at quite high levels of average coverage, the vast majority of errors are predicted to come from the ends of reads in single-coverage regions of an assembly. These calculations are based on a distribution of single-read quality scores estimated empirically from the tupBel1 (Northern Tree Shrew) assembly (see Materials and Methods). For comparison, points corresponding to the error rates in Table 2 are shown (plus symbols), with tupBel1 in black. Departures from the theoretical predictions are partially explained by departures from the assumption of Poisson-distributed read depths (Table S4).

We devised a simple theoretical model to explore the relationship between sequencing error and read coverage as a function of the average coverage. The model assumes Poisson-distributed read depths, independence of quality scores across reads, and quality scores that accurately predict error rates, and it relies on an empirically determined distribution of quality scores from single reads (see Materials and Methods). Because it uses quality scores as a proxy for true error rates, the model does not distinguish between basecall and indel errors, but aggregates them together. This model indicates that the overall sequence quality, expressed in *phred* units as −10 log_10_ *p*(*error*), should increase nearly linearly with the expected coverage over the range of interest, such that the error rate will be approximately halved by each additional 1× of sequencing (Figure 3). Moreover, it predicts that this increase in quality will be almost completely driven by decreases in single coverage regions in the assembly, which contribute the vast majority of the error. Indeed, the model predicts that 98.9% of errors will come from single coverage regions in a 2× assembly. This fraction remains high as the average coverage (*λ*) grows larger, with values of 96.8% for *λ* = 6, 94.8% for *λ* = 10, and 92.2% for *λ* = 15. Furthermore, nearly all of the errors in these single-coverage regions can be attributed to a relatively small fraction of bases with low quality scores. For example, the 13.7% of single-read bases with *q*<20 are expected to contribute 93.5% of the error in single coverage regions, or 92.5% of all errors at *λ* = 2. These low-quality bases occur preferentially at the ends of reads, with 90% occurring either 50 bp from the 5′ end or 300 bp from the 3′ end of a read (Figure S1). Thus, consistent with intuition, the model predicts that the vast majority of sequencing errors will be contributed by bases at the ends of reads in single-coverage regions of an assembly, but somewhat surprisingly, it shows that this property holds even for quite large values of *λ*.

### Implications for phylogenomics

**Figure 4:**
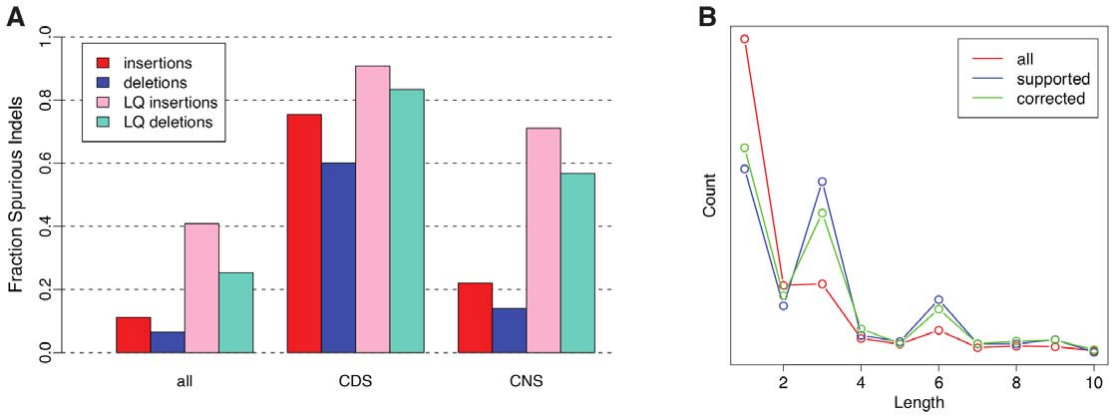
Error rates and length distributions for lineage-specific indels. (A) Fractions of apparent lineage-specific indels that are spurious, as estimated from ENCODE/2× alignments. Shown are median values over all 2× species after the polymorphism correction, for all sites and for only those sites within coding (CDS) or conserved noncoding (CNS) regions. Notice that insertions are substantially more likely than deletions to be spurious, because true deletions occur at higher rates than true insertions [29], while insertion and deletion error rates are comparable. LQ categories are the subset of indels with low quality (<25). (B) Distribution of indel lengths in coding regions for mouse lemur as compared to human, using dog as an outgroup. The red line (all) represents all indels inferred from the 2× assemblies, while the blue lines (supported) represents a subset of indels that could be validated by comparison with the ENCODE mouse lemur sequence. The green line (corrected) shows the distribution for the 2× assembly after automatic sequencing error mitigation (SEM) was applied using a quality score threshold of <25. Notice that the pronounced period-of-three pattern in the supported indels is nearly lost in the unprocessed data due to sequencing error, but is mostly recovered by the SEM procedure. The effect is similar for the other 2× assemblies.

**Figure 5:**
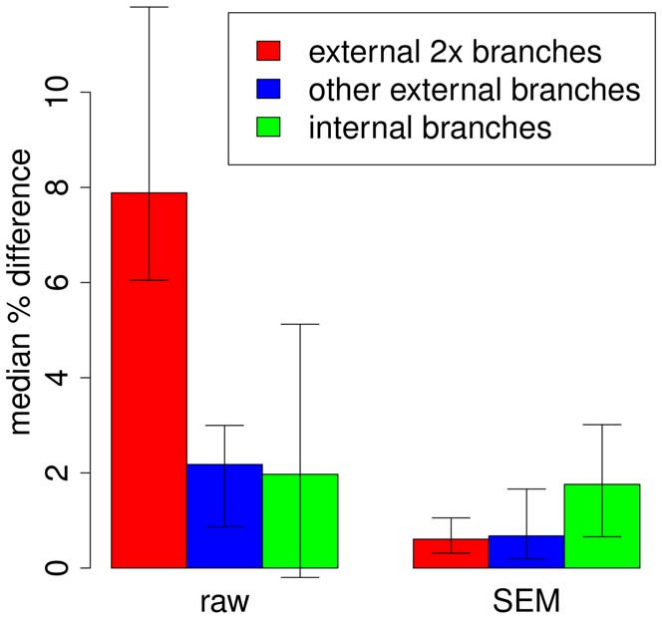
Estimated percent error in indel rates. Results are shown for the raw and error-mitigated (SEM) data sets. Indel rates were inferred using a probabilistic indel model and an expectation maximization algorithm (Methods S1), and percent error was estimated by comparing the rates for each data set with ones based on the highest quality sites only (*q*≥45). The median across the branches within each group is shown, with error bars indicating upper and lower quartiles. Results are shown for three groups of branches from the 32-species phylogeny: external branches corresponding to 2× species (red), all other external branches (blue), and internal branches (green). The indel rates fir the external 2× branches are overestimated by nearly 8% with the raw data, but that the SEM procedure eliminates most of this effect. There is also a substantial amount of overestimation associated with non-2× external branches stemming from lower-quality draft assemblies, such as the chimpanzee, and the SEM procedure effectively reduces this error as well.

To gain insight into the implications of sequencing error in phylogenomic applications, we performed a series of simple, descriptive analyses. First, we asked what the average total number of errors would be in a typical coding exon, allowing for different exon-specific error rates per species and the effects of missing data in the aligments (see Materials and Methods). We found that a protein-coding exon of 120 bp (approximately the median length) will contain an average of 4.0 spurious indels and 5.8 miscalled bases, across all of the low-coverage genomes present (for illustration, see Figure S2). Thus, while the error rate per low-coverage genome is modest, the aggregate error in a multiple alignment of twenty such genomes can be substantial. Next, we examined how the probability of error changes when conditioning on properties of interest, focusing on the case of indels that appear to be lineage-specific (LS)—that is, indels present in a 2× sequence but not in the other sequences according to the multiple alignments (see Materials and Methods). We found overall, among apparent LS indels, 11.1% of insertions and 6.5% of LS deletions are spurious (Figure 4A). However, in coding regions, 75.4% of LS insertions and 60.1% of LS deletions are spurious, owing to the substantially reduced rates of true indels in these regions. The error rates for LS indels in conserved noncoding regions are also elevated, although less so than in coding regions, at 22.1% (insertions) and 14.0% (deletions). Third, we examined the length-distributions of LS indels in coding regions, considering (a) all LS indels in the ENCODE regions and (b) only those that are supported by the comparative-grade ENCODE sequences. We found that indels supported by high quality sequence display a clear preference for multiple-of-three lengths (which preserve the reading frame), but this periodicity in the length distribution is almost completely erased by sequencing error in the raw 2× assemblies (Figure 4B). Finally, we examined the impact of sequencing error on inferred rates and patterns of insertion/deletion and substitution on the mammalian phylogeny. Using a probabilistic model of insertion and deletion and an expectation-maximization (EM) algorithm (Methods S1), we found that the raw 2× assemblies produce substantially inflated estimates of indel rates compared with estimates based on high-quality sequences only, particularly for external branches of the phylogeny (Figure 5). A similar analysis indicated that, overall, substitution rates are relatively unaffected by sequencing error (data not shown), but that error can inflate the ratio of nonsynonymous to synonymous substitution rates (*d*_N_/*d*_S_) in protein-coding genes by as much as ∼10% (Methods S2 and Table S3).

Taken together, these descriptive analyses demonstrate that sequencing error has clear effects on the apparent rates and patterns of both substitutions and indels, and that it has a particularly pronounced effect for indels. Sequencing error may have important consequences not only on estimates of absolute rates of mutations, but on phylogenomic analyses that depend on the relative rates of events, as in the identification of novel functional elements and in predictions of positive selection [20], [21].

### Sequencing error mitigation

The highly localized nature of sequencing error, with a large majority of errors coming from a small minority of bases, raises the possibility of using automatic methods to mitigate the effects of error in downstream analyses, a procedure we refer to generically as *sequencing error mitigation* (SEM). In this section, we explore several alternative strategies for SEM.

#### Base-call error

Our general approach to base-call error is to mask erroneous bases by converting them to “N”s. We do not attempt to revise the base-calls at positions predicted to be miscalled because only weak information about their true identity is available. Our baseline strategy is simply to apply a threshold to the nominal quality score for each base (as reported for the genome assembly), masking any base with a score below the designated threshold. By considering no sources of data other than the sequence itself, this strategy has the advantage of being easy to interpret and avoiding complex biases in downstream analyses.

We measure the false positive rates (FPRs) and true positive rates (TPRs) of base-masking decisions as a function of the quality score threshold, where the FPR is defined as the fraction of correctly called bases that are unnecessarily masked, and the TPR is defined as the fraction of miscalled bases that are masked. We also measure the positive predictive value (PPV), or the fraction of masked bases that actually were miscalled, and hence, were correctly masked. These quantities are measured with respect to the ENCODE data in regions covered by high-confidence ENCODE/2× alignments, then corrected for polymorphisms (Materials and Methods).

**Figure 6:**
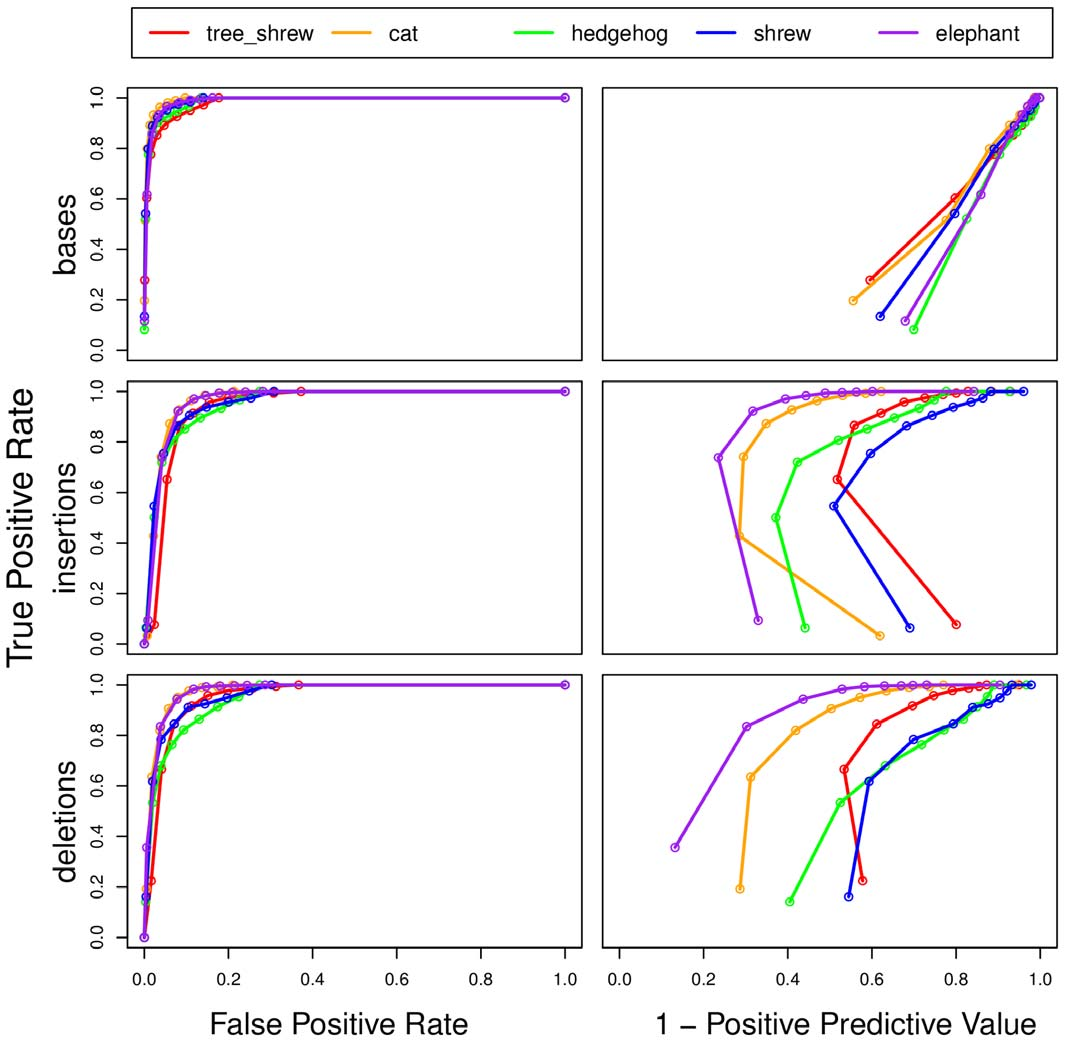
Receiver operating characteristic (ROC) curves for sequencing error mitigation. On the left, false positive rates (fractions of non-errors incorrectly masked or imputed) are plotted versus true positive rates (fractions of errors correctly masked or imputed) as the quality threshold is varied (see text). On the right is a similar plot with 1 minus the positive predictive value (indicating the fraction of masking or imputation decisions incorrectly undertaken) in place of the false positive rate. Results are shown for five representative 2× assemblies, with separate plots for basecall (top), insertion (middle), and deletion (bottom) errors. The results shown here reflect simple thresholding of quality scores (see also Figure 7).

We find that this simple masking strategy allows large fractions of miscalled bases to be masked with relatively low FPRs. For example, at a quality threshold of 10, roughly 80% of miscalled bases can be eliminated at a FPR of ∼1%, with slight differences between genome assemblies (Figure 6). By choosing a threshold of 20, a TPR of ∼90% and a FPR of 5% can be achieved. However, because error rates are fairly low even for low quality scores, absolute FPRs of 1–5% may still result in large amounts of “over-masking”. Indeed, at a threshold of 10, with a TPR of ∼80%, the PPV is ∼10%, meaning that 9 correct bases must be unnecessarily masked for every miscalled base that is appropriately masked. At a threshold of 20 (TPR 90%) the PPV is only ∼4%.

**Figure 7:**
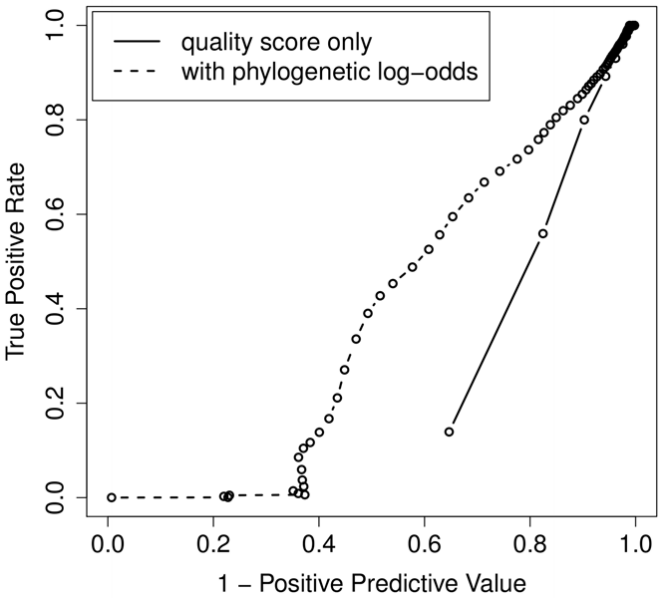
ROC-like plot for logistic regression-based SEM. Results are shown for one species, the bushbaby (see Figure S6 for others). See Figure 6 for comparison. The solid curve represents simple thresholding on quality score and the dashed line represents the use of logistic regression with both quality scores and phylogenetic log-odds scores as covariates (see Materials and Methods).

We attempted to improve these results by using a regression-based method to predict whether or not to mask each base. Specifically, we trained a logistic regression classifier on a subset of the ENCODE data, making use of several relevant covariates, including the aligned bases from other species, the local G+C content and the quality scores of neighboring bases (see Materials and Methods), and then tested the method on a separate portion of the ENCODE data. To summarize the information from aligned bases, we included as one of the covariates a log-odds score based on a phylogenetic error model, which indicates how much more likely an observed base is under a model of sequencing error vs. a model that allows for true nucleotide substitutions only. This method did significantly improve performance (Figure 7). For example, in the case of bushbaby, at a TPR of 50% the regression-based method roughly doubled the PPV, from ∼20% to ∼40%. However, the improvement was more pronounced at midrange TPRs than at higher TPRs, which are of greater practical interest. For example, at a TPR of 80% in bushbaby the PPV is increased only from ∼10% to ∼15%. The degree of improvement also varied considerably among species, depending on the availability of cross-species alignments, branch lengths in the phylogenetic tree, and other factors. Because such dependencies may lead to difficult-to-interpret biases in downstream analyses, and because the regression-based method is computationally expensive, we selected the simpler, quality-score thresholding method as our default base-masking strategy.

#### Indel error

We address indel error using an imputation strategy rather than a masking strategy. Our general approach is to identify indels likely to be spurious, based on the multiple alignments and quality scores, then to “correct” these indels by filling in false deletions with “N”s, or excising false insertions. Specifically, we identify indels that can only be parsimoniously explained by lineage-specific insertion or deletion events, using an exact dynamic-programming algorithm (see Materials and Methods). If these apparent lineage-specific indels are supported only by low-quality sequence, we conclude that they are more likely to result from sequencing errors than from true evolutionary events, and we revert the sequence to the inferred ancestral indel state. Only lineage-specific indels are corrected, because indels that are shared between species would require multiple coinciding errors to explain, and therefore are much less likely to be erroneous.

As with our basecall error mitigation strategy, we evaluated the TPR, FPR, and PPV of this approach as the threshold for low-quality sequence was varied. In this case, the TPR is the fraction of spurious indels that were properly “corrected”, the FPR is the fraction of true indels that were unnecessarily edited, and the PPV is the fraction of corrections that improve the quality of the assembly. These measures consider only “eligible” indels—that is, ones predicted to be lineage-specific by the parsimony algorithm—and therefore can range from 0 to 1. We experimented with various measures for identifying “low-quality” sequence (see Materials and Methods), but settled on the relatively simple measure of the minimum quality score observed within a 5 base pair distance of the indel.

In terms of absolute TPRs and FPRs, the indel algorithm appears to perform slightly worse than the base masking algorithm Figure 6. For example, to obtain a TPR of 80%, a FPR of ∼6% must be tolerated for either insertions or deletions. However, this difference is misleading because false positives are counted in quite different ways in the two cases—for basecalls, they are counted as fractions of all correct bases (i.e., the vast majority of bases), while for indels they are fractions of correct lineage-specific indels (a much smaller collection). Indeed, when measured in terms of PPV, error mitigation is substantially better for indels than for basecalls, with values of roughly 30–80% at a TPR of 80%. (Notably, there is considerable variation across species, because the algorithm depends on the position of each species in the phylogeny.) For a typical genome, say tree shrew, it is possible to correct 80% of eligible indels by altering only slightly more than twice as many cases as should be altered (i.e., with a PPV of 50%). The favorable PPV for indels results from the fact that true indel rates are quite low (roughly 20-fold lower than substitution rates; [29]), yet indel error rates are comparable to those for basecalls, typically differing only by a factor of 4–5 (Table 2). Similarly, the PPVs for insertions are somewhat better than those for deletions, because the true deletion rate is larger than the true insertion rate [29].

A limitation of this approach is that many indel errors are not eligible for correction. For example, spurious indels in a human-specific *Alu* insertion will not be able to be corrected, because alignments to orthologous sequences in other mammalian species will not be available. Other spurious indels will not be inferred as lineage specific, due to alignment errors or failures of the parsimony algorithm. We find that the fraction of spurious indels that are eligible for correction ranges from about 1/3 to more than half, depending on the species in question (Figure S3). Of the ineligible indels, most are in the alignments but are not inferred to be lineage-specific, although in some species, such as hedgehog, the situation is reversed—possibly because of long branches in the phylogeny or large numbers of transposons. The aspect of the approach may improve substantially as more species are sequenced and alignment algorithms improve.

#### Consequences in phylogenomics

To shed light on the impact of SEM in downstream analyses, we examined four representative applications. For this analysis, we used simple quality score thresholds of <20 for base-masking and <25 for indel imputation (corresponding to TPRs of ∼88% and ∼96% in the experiments above).

We first considered the length distribution of indels, which was shown above to be strongly influenced by indel error (Figure 4B). We found that SEM significantly improved the inferred length distribution, bringing it nearly in line with the distribution estimated from the high-quality ENCODE data. In particular, SEM eliminated a large number of non-multiple-of-three-length indels, including many of length one, and caused the pronounced period-of-three signal, which was nearly lost in the raw 2× data, to re-appear. Second, we re-estimated indel rates on the branches of the phylogenetic tree for these species, as described above, after applying SEM to the alignments. We found that SEM substantially reduces the inflation in indel rates that arises from sequencing error (Figure 5). In our third analysis, we examined the impact of SEM on estimates of *d*_N_/*d*_S_ ratios, which, as discussed above, can be inflated by error. Again, SEM was effective in eliminating most of the upward bias in *d*_N_/*d*_S_ estimates (Table S3). Finally, we examined the impact of SEM in comparative functional element detection, using a recently developed comparative exon-finding method called CONGO [30]. After retraining the method with the SEM-processed alignments, CONGO's exon-level specificity with respect to genes from the RefSeq, ENSEMBL, UCSC, and GENCODE gene sets increased modestly yet significantly, from 89.9% to 90.5% (p = 1.2×10^−9^, χ^2^ test), while the sensitivity increased slightly, from 74.3% to 74.4% (see Methods S3 and Table S4 for full details). While these increases were modest in absolute value, the increase in specificity held consistency across all chromosomes in the human genome, except for the Y chromosome (Table S5).

## Discussion

Since the advent of large-scale DNA sequencing, sequencing error has periodically emerged as a research topic of primary interest in genomics [4], [7], [8]. In this article, we have examined the issue of sequencing error with respect to twenty newly available low-coverage (2×) mammalian genome assemblies. This data set will ultimately be supplanted by draft genomes, and is therefore of transient interest. Nevertheless, it is likely to dominate the field of mammalian comparative genomics for the next few years. Our focus in this article has been to characterize the sequencing error in these data, to examine its implications for phylogenomic analysis, and to explore simple strategies for mitigating the effect of error in certain applications of interest.

While these 2× genome assemblies are generally of high quality, they do display significant amounts of error. Base-call errors occur at rates of ∼1–3 per kb, and indels at ∼0.5–1.0 per kb. The overall error rate in these sequences, at an average of 2.5 errors per kb, is comparable to the average rate at which nucleotide differences occur between individuals of a typical mammalian species (π). Importantly, these errors are highly localized, with ∼4% of bases contributing roughly 90% of base-call errors and 75% of indel errors. Sequencing error appears to come primarily from the ends of reads in single-coverage regions of the assembly. The impact of this error in downstream analyses depends on the relative rates of error and real events of interest. Some mutations—such as lineage-specific insertions in protein-coding sequences—are sufficiently rare that apparent mutations in the 2× alignments are considerably more likely to be spurious than real. Accordingly, certain patterns of interest in comparative genomics—such as the length distributions of short indels in coding regions—can be strongly skewed by sequencing error. At the same time, sequencing error does not severely impact all analyses of interest. It had only a minor effect on inferred substitution rates, and its effects on *d*_N_/*d*_S_ estimates and on a state-of-the-art comparative exon-finding method were more pronounced but still limited.

Automatic methods for sequencing error mitigation (SEM) can reduce the effects of error to a degree. Indel imputation, in particular, is effective at reducing the number of spurious lineage-specific indels, and can substantially reduce the distortion of indel length distributions and numbers of lineage-specific indels imposed by error. As a result, SEM could, in principle, produce significant improvements in downstream phylogenomic analyses, especially ones dependent on indel rates and patterns. In our experiments, it did significantly improve performance in comparative exon prediction. Performance improvements of ∼1% in exon-prediction, while not dramatic, are difficult to achieve without major modeling or algorithmic innovations, and it is notable that they were achieved in this case simply by preprocessing the input to the algorithm. At the same time, this example demonstrates that sophisticated machine-learning methods can effectively compensate for the effects of sequencing error in functional element prediction, and may only benefit slightly from a separate SEM step. SEM may be of greater value in straightforward estimation of mutation rates or with simpler methods for functional element prediction.

Several general issues arise in deciding on an appropriate SEM strategy. We settled on quite simple methods, based on thresholding of quality scores, for efficiency and ease of interpretation. However, our experiments with regression-based methods suggested that the use of additional covariates can significantly improve performance. It may be possible to improve performance further by making use of a richer set of features in a more sophisticated classification framework, including, say, the primary traces for sequencing reads and the full multiple alignment of reads used by the assembler [19]. A second question we faced was whether to mask bases that are predicted to be erroneous or to impute specific values for them. In general, masking is preferable in that, when making unnecessary “corrections”, it simply discards data rather than introducing new errors. For indels, however, we found masking impractical and instead settled on an imputation strategy. The disadvantage of this approach is that it can produce biases of its own, for example, by reducing the apparent indel rates on terminal branches of the phylogeny. A third question is whether to make use of alignments with other species in SEM. Alignments are clearly informative about error but their use has certain drawbacks: they cannot be used uniformly across the assembly (because some regions do not align), they are not equally informative about all species (because of differences in phylogenetic position), and their usefulness depends on the accuracy of the alignment methods. We have used alignments for indel imputation, where they are particularly informative, but not for base masking. However, alternative approaches may be preferable for certain applications.

Our theoretical model suggests that regions of single read coverage in the assembly are dominant in determining overall error rates. Interestingly, this property is predicted to hold even at quite high coverage—for example, more than 90% of errors are predicted to derive from regions of single read coverage at 15× coverage (Figure 3). There are two major ways in which simplifying assumptions of the model could impact our calculations. First, the model may underestimate the error rates at bases covered by multiple reads, by assuming independence across reads in computing aggregate quality scores. Our empirical data suggests some underestimation of error rates does occur at large quality scores (*q*≥30) (Figure 1). However, because smaller quality scores (*q*<20) are dominant in determining error rates, we expect this underestimation to have only a minor effect on our conclusions. Second, departures from the assumption of Poisson-distributed read depth may lead to an excess of single-read regions at high average coverage (see Figure S4). This would reduce the rate of increase in expected sequence quality (*Q* ^*^) as average coverage (*λ*) increases, making *Q* ^*^ sublinear in *λ* (see, for example, the point in Figure 3 at 6.8× coverage, representing guinea pig). However, it should *increase* the fraction of errors coming from regions of single read coverage at large λ, making them more like those for small λ. Thus, we expect the conclusion that regions of single read coverage are dominant even at large *λ* to be robust to our modeling assumptions. This property should be borne in mind when interpreting claims that the vast majority of bases in a genome assembly have high quality—for example, that 98% of bases have quality scores of at least 40 [12], [31]. While this “mostly very high quality” property is undoubtedly useful in some respects, it may still be compatible with fairly high overall error rates, because few bases with high error rates can make a strongly disproportional contribution to the overall average. Mathematically, this can be understood by noting that the expected sequence quality *Q* ^*^ is determined not by the arithmetic mean of the basewise quality scores, but by a generalized *f*-mean with 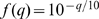, which gives large weight to small values of *q*.

While most of the genome sequences considered here will eventually be replaced by higher coverage assemblies, other projects parallel this one in certain respects. For example, low-coverage sequencing is underway for several fungi and unicellular eukaryotes and for three East African cichlid fishes (http://www.genome.gov/10002154). Metagenomic projects also tend to produce low-coverage data for each organism of interest, because of the diversity of source material, and some large-scale within-species sequencing projects—including the 1000 Genomes Project—have used relatively low-coverage sequencing strategies (say, 4×) for SNP discovery. In addition, as shown by our theoretical analysis, consideration of sequencing error can continue to be important even with high-coverage assemblies, particularly for applications in comparative or population genomics. For these reasons, there is a need to integrate better models of sequencing error into a wide variety of sequence analysis methods, including alignment, assembly, gene finding, and phylogenetic modeling. Notably, improved standards at the assembly level would help to enable the development of error-aware sequence analysis tools. Ideally, assembly quality scores would be produced in a uniform manner, read-depth information would be available for each assembled base, and full read tiling paths would be obtainable for assembled genomes.

## Materials and Methods

### 2× Assemblies

The twenty-two new genome assemblies (Table 1) were assembled using a combination of the ARACHNE assembler [32], [33] and a novel “assisted” assembly method that used alignments with the human, mouse, and dog genome assemblies to improve long-range contiguity. They were then aligned with each other and with existing finished or draft-quality genome assemblies using the multiz program [34] and related tools from the UC Santa Cruz alignment pipeline [35]. The final multiple alignments, represented in Multiple Alignment Format (MAF), consisted of 29 eutherian mammals plus three non-eutherian outgroup species (opossum, chicken, and tetraodon), for a total of 32 species. For convenience in analyzing sequence alignments and quality scores together, the MAF files were augmented to include basewise quality scores, in a reduced representation. Specifically, each raw quality score *q* was mapped to an integer 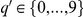 using the formula 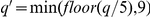 (see http://genome.ucsc.edu/FAQ/FAQformat). Thus, the raw quality scores were captured with a resolution of 5 *phred* units, except that all scores ≥45 were considered equal. (Notably, the ARACHNE assembler sets a maximum quality score of 50.) This reduced representation was used in our subsequent analyses.

### Alignment with ENCODE sequences

BLASTZ [36] was used to align each scaffold of the 2× genomes to the corresponding high-quality ENCODE sequence for that species, for every species for which ENCODE sequence was available. This was followed by a step of chaining and netting to produce a single-coverage alignment for each 2× assembly. After a close inspection of these alignments, we realigned the sequences using LASTZ (http://www.bx.psu.edu/miller_lab) to reduce alignment error. These alignments were then passed through a final set of chaining, netting, synteny, and reciprocal best filters.

### Polymorphism correction

Let *d_esq_* be the rate at which differences suggesting events of type *e* occurred in the 2×/ENCODE alignments for species *s* in regions having 2× quality score *q*. Let *d_esQ_* represent the rates for the highest quality regions (*q*≥45). Polymorphism-adjusted error rates were estimated, in a quality score-specific manner (for *q*<45), as *r_esq_* = *d_esq_−d_esQ_*. When assessing the performance of sequencing error mitigation, a fraction *d_esQ_*/*d_esq_* of differences were assumed to be polymorphisms for each *q*, *s*, and *e*. These fractions were used to compute the expected number of masked sites that were polymorphic, which was added to the count of false positives and subtracted from the count of true positives. Similarly it was used to compute the expected number of unmasked sites that were polymorphic, which was added to the count of true negatives and subtracted from the count of false negatives.

### Theoretical model

Our theoretical model for the relationship between read depth and sequencing error assumes (1) that the read depth at each base is Poisson-distributed with mean *λ* equal to the average coverage [37]; (2) that true error rates are well predicted by nominal quality scores (see Figure 2); (3) that quality scores are independent across aligned reads at each position in the assembly, so that the joint distribution of quality scores at a position *i* with read depth *x*, 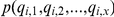, is equal to a product of marginal distributions, 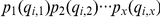, where *p*_1_ (*q*) is the probability of observing a quality score *q* in an individual read; and (4) that a quality score for an assembled base can be accurately expressed as a sum of the quality scores in the individual reads (as assumed by ARACHNE). Under these assumptions, several quantities of interest can be computed easily for a given distribution *p*_1_ (*q*), which can be estimated empirically using data from the public trace archives (see below). For example, the overall distribution of quality scores for an assembly with average coverage *λ* is given by
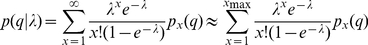
(1)where *p_x_* is an *x*-wise convolution of *p*_1_, which can be computed by a simple recursive calculation, and *x*_max_ is large relative to *λ* (say, the O.999 quantile of the Poisson distribution with mean *λ*). The expected overall error rate in the assembly, expressed in *phred* units, is:
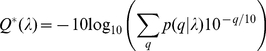
(2)Further details are given in Methods S4.

To determine *p*_1_, we examined the empirical distributions of single-read quality scores for fourteen 2× assemblies (as well as one 7× assembly), using random samples of reads from the NCBI Trace Archives http://www.ncbi.nlm.nih.gov/Traces/. For the most part, these distributions were similar across species (Figure S5), and we selected one that was fairly typical of the group for our analysis (tupBel1). In estimating *p*_1_, we excluded reads and trimmed portions of reads that were not incorporated into the tupBel1 assembly.

### Exonic error rates

To estimate the error rates per exon, we identified 2,668 nonoverlapping coding (CDS) exons within the ENCODE regions from the UCSC Genes set, and counted differences between the aligned 2× and ENCODE sequences within these exons. These counts were corrected for polymorphisms in a quality score-specific manner, as above, but using CDS-specific estimates of polymorphism rates. The counts were multiplied by 20/14 to adjust for the fact that ENCODE sequences were available for only fourteen of twenty species, and, finally, converted to rates per 120 bp exon. Notably, this calculation reflected the reduction in error due to incomplete coverage of the 2× assemblies (we did not compensate for this reduction).

### Base masking

In the simplest case, each base *x_i_* was masked (changed to an “N”) if, and only if, the corresponding quality score *q_i_*<*T*, where *T* is a threshold (typically, *T* = 20). In the case of logistic regression, *x_i_* was masked if, and only if, 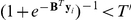, where **B** is a vector of regression coefficients, **y** *_i_* is a vector of local covariates, including the local G+C content, the quality scores of flanking bases, and a phylogenetic log-odds score (see below), but found a combination of the local quality score *q_i_* and the log-odds score to perform best. The regression coefficients **B** were estimated from half of the ENCODE data, and accuracy was assessed on the remaining half. Parameter estimation was accomplished with the *glm* function in R.

The log-odds score for base *x_s_* from species *s* in alignment column *X* is defined as 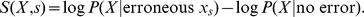 The quantity 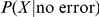 is computed by applying Felsenstein's algorithm [38] to *X*, using a phylogenetic model estimated from fourfold degenerate sites (2× Mammals Consortium, in prep.). To compute 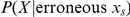, we introduce a species-specific error transition matrix 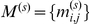, such that 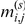 is the probability that the true base *i* is erroneously represented as a *j* in the 2× sequence for species *s* (thus, 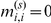 for all *i*,*s*). We then calculate:
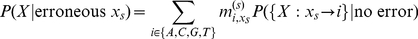
(3)where 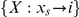 denotes a version of *X* in which *x_s_* is replaced with base *i*. This calculation assumes that the probability of multiple errors in a single column X is negligible. The matrix *M* ^(*s*)^ is estimated empirically from the 2×/ENCODE alignments, using separate data for training and testing. To accommodate variation in substitution rates, we introduce a branch-length scaling factor and optimize it (separately) for both the error and error-free models, using methods recently described elsewhere [39]. As a result, an apparent substitution at a conserved site is considered more likely to be an error than a similar substitution at a less conserved site.

### Indel imputation

To identify lineage-specific (LS) indels, we applied a Sankoff-like parsimony algorithm [40] to a reduced representation of each *indel region* (IR) in the multiple alignments. An IR is a maximal sequence of alignment columns having at least one gap character each and flanked by gapless columns. To simplify these regions and focus on short indel events, we considered only gaps of ≤10 bp, allowing for gaps between MAF blocks provided that they connected contiguous bases in a sequence. We represented the IRs as matrices of 0s (gaps) and 1s (bases). Because all indel events were given equal cost regardless of length, the starting matrix could be compressed by replacing each sequence of identical columns with a single representative [41]. Associated with a matrix of width *n* were 2*^n^* possible indel states. To find parsimonious labelings of ancestral nodes by indel states, we defined a 2*^n^*×2*^n^* matrix, *W* = {*w_i_*,*_j_* }, of state-transition costs, such that *w_i_*,*_j_* equaled the minimum number of indel events for a transition from state *i* to state *j*. Using *W* as a cost matrix for each branch, we applied the max-product algorithm [42] to the phylogeny and enumerated all states at each node that were consistent with parsimonious labelings. Cases in which a leaf was represented by a 0 but its parent was only allowed a 1 by parsimony were inferred to be LS deletions. Similarly, cases in which a leaf was represented by a 1 but its parent was only allowed a 0 by parsimony were inferred to be LS insertions. LS events were inferred column-by-column, so it was possible that only part of an IR would be considered lineage-specific. This was important because, with large number of species, multiple overlapping LS events are not unusual, and in many cases they can be unambiguously disentangled (the are effectively independent). For reasons of computational efficiency, IRs with n>10 (0.36% of all IRs) were ignored. As discussed in the text, once an LS indel was identified in a sequence *s*, it was “corrected” (reverted to the ancestral state) if, and only if, it corresponded to low-quality bases in *s*. As a measure of indel quality, we used the minimum quality score in the five flanking bases on either side of the indel and in any inserted bases. We experimented with several alternative measures of quality but none performed better than this simple measure. We emphasize that we did not attempt to revise the alignment in this procedure; this would have required a much more complex and computationally expensive algorithm.

Details concerning indel rates, *d*_N_/*d*_S_ ratios, and the CONGO exon predictions are provided in Methods S1, S2, S3.

## Supporting Information

Figure S1 **Distribution of low-quality bases along a read.** Empirical cumulative distribution functions showing fractions of bases with *phred* scores <20 that occur at various distances from the 5′ (solid line) and 3′ (dashed line) ends of each read. Low quality bases are strongly concentrated within 50 bases of the 5′ and 300 bases of the 3′ end of each read. Distributions are based on 9,905 randomly chosen tree shrew reads, which were chosen conditional on inclusion in the final assembly. The position on the x-axis is read position after the trimming performed a pre-processing step by the assembler.
(TIF)

Figure S2 **UCSC Genome Browser screen shot showing potential spurious indels in multiple alignment.** Screen shot from the UCSC Genome Browser [6], showing the multiz-based genome-wide alignments—including the twenty low-coverage genome assemblies—for a coding exon of the *caveolin I* (*CAV I*) gene. Five apparent lineage-specific frame-shifting deletions in the mouse lemur sequence are shown. Comparative-grade sequence for mouse lemur from ENCODE region ENm001 indicates that these deletions reflect errors in the 2× assembly, rather than genuine deletion events in the evolution of the mouse lemur. This possibility is further supported by low phred scores in the 2× assembly (values of 3–23 flanking the deletions, and a mean score of 14 in the region), evidently reflecting single read coverage. Spurious indels of this kind can have numerous damaging consequences in phylogenomic analyses, for example, by distorting inferred indel rates, by causing comparative gene finders to fail, or by producing false signatures of accelerated lineage-specific evolution. While it is somewhat unusual to observe a tight cluster of five indels in one sequence, as in this case, spurious indels in coding regions are generally common in the 2× alignments (see text).
(TIF)

Figure S3 **Fraction of indels in the 2×/ENCODE alignments which can be mapped to the 44-way alignment.** The black slices represent the fraction of these indels in which no corresponding indel appears in the 44-way alignment, and may be explained by polymorphism. The blue slices appear as lineage-specific indels in the 44-way alignment, and are candidates for error mitigation when the quality score is sufficiently low. The red slices appear as non-lineage-specific indels and cannot be addressed by our method. These may be explained by alignment error or old polymorphism segregating in several species.
(TIF)

Figure S4 **Empirical cumulative distribution functions (CDFs) for read depths in various assemblies.** The distributions expected under comparable theoretical (Poisson) model are shown for comparison. Read depth was estimated from the assembly.reads file produced by the ARACHNE assembler. This file contains information about approximate placement of each read in the assembly. Because these placements are not exact, the depths should also be considered approximate.
(TIF)

Figure S5 **Empirical cumulative distribution functions (CDFs) for single-read quality scores in various assemblies.** This figure is based on a random sample of ∼10,000 reads per assembly, and only represents the portion of each read which is used in the assembly. Tree shrew (shown in bold) was chosen as a representative species whose distribution was used in our model for coverage and error.
(TIF)

Figure S6 **Performance of base masking algorithms which use linear-regression framework, for four species.** The dark black lines show the baseline results using only quality scores. In red are the results where regression is performed on the quality scores and a phylogenetic odds ratio comparing a model with error to a model without error. In purple, blue, and green, respectively, are results which use both quality score and GC percentage in a 10 bp window, the minimum quality score in a 10 bp window, and the mean quality score in a 10 bp window. The only factor which seems to have a positive effect on the prediction is the phylogenetic odds ratio.
(TIF)

Table S1 **Species nomenclature.**
(PDF)

Table S2 **Summary of alignments of 2× genomes and corresponding ENCODE sequences.**
(PDF)

Table S3 **Estimates of** ***d*** **_N_/** ***d*** **_S_ for chr22 genes and four primates.**
(PDF)

Table S4 **CONGO performance with and without SEM.**
(PDF)

Table S5 **CONGO performance by chromosome.**
(PDF)

Methods S1 **Estimation of indel rates.**
(PDF)

Methods S2 **Estimation of** ***d*** **_N_/** ***d*** **_S_.**
(PDF)

Methods S3 **CONGO exon-finding analysis.**
(PDF)

Methods S4 **Theoretical model.**
(PDF)
