# Supplementary figures and images for: Error and Error Mitigation in Low-Coverage Genome Assemblies
Source: PLoS One. 2011 Feb 14;6(2):e17034. doi: 10.1371/journal.pone.0017034 (PMC3038916; doi:10.1371/journal.pone.0017034)

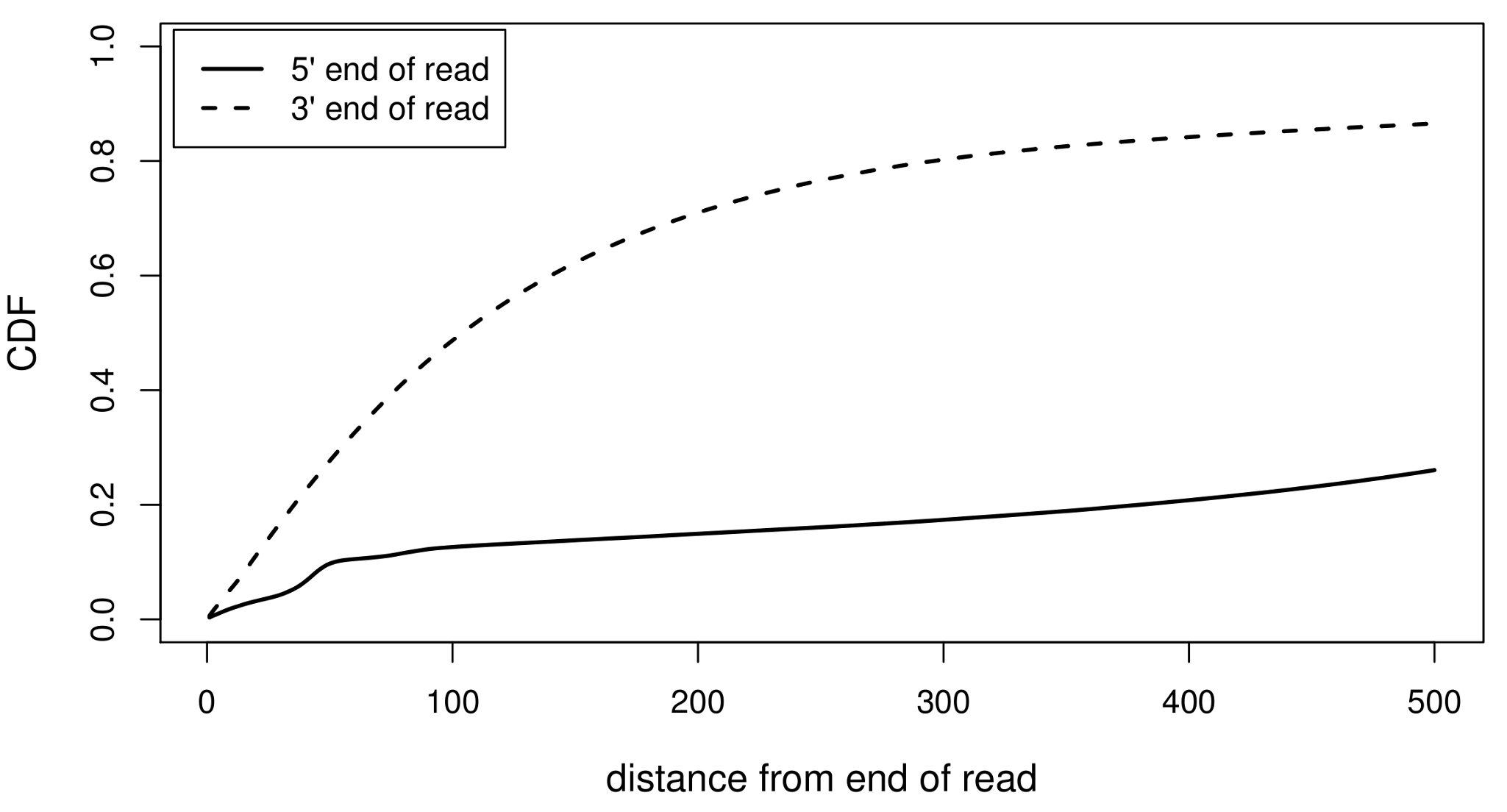

Supplement: Figure S1 — Distribution of low-quality bases along a read. Empirical cumulative distribution functions showing fractions of bases with phred scores <20 that occur at various distances from the 5′ (solid line) and 3′ (dashed line) ends of each read. Low quality bases are strongly concentrated within 50 bases of the 5′ and 300 bases of the 3′ end of each read. Distributions are based on 9,905 randomly chosen tree shrew reads, which were chosen conditional on inclusion in the final assembly. The position on the x-axis is read position after the trimming performed a pre-processing step by the assembler. (TIF) [file pone.0017034.s001.tif]

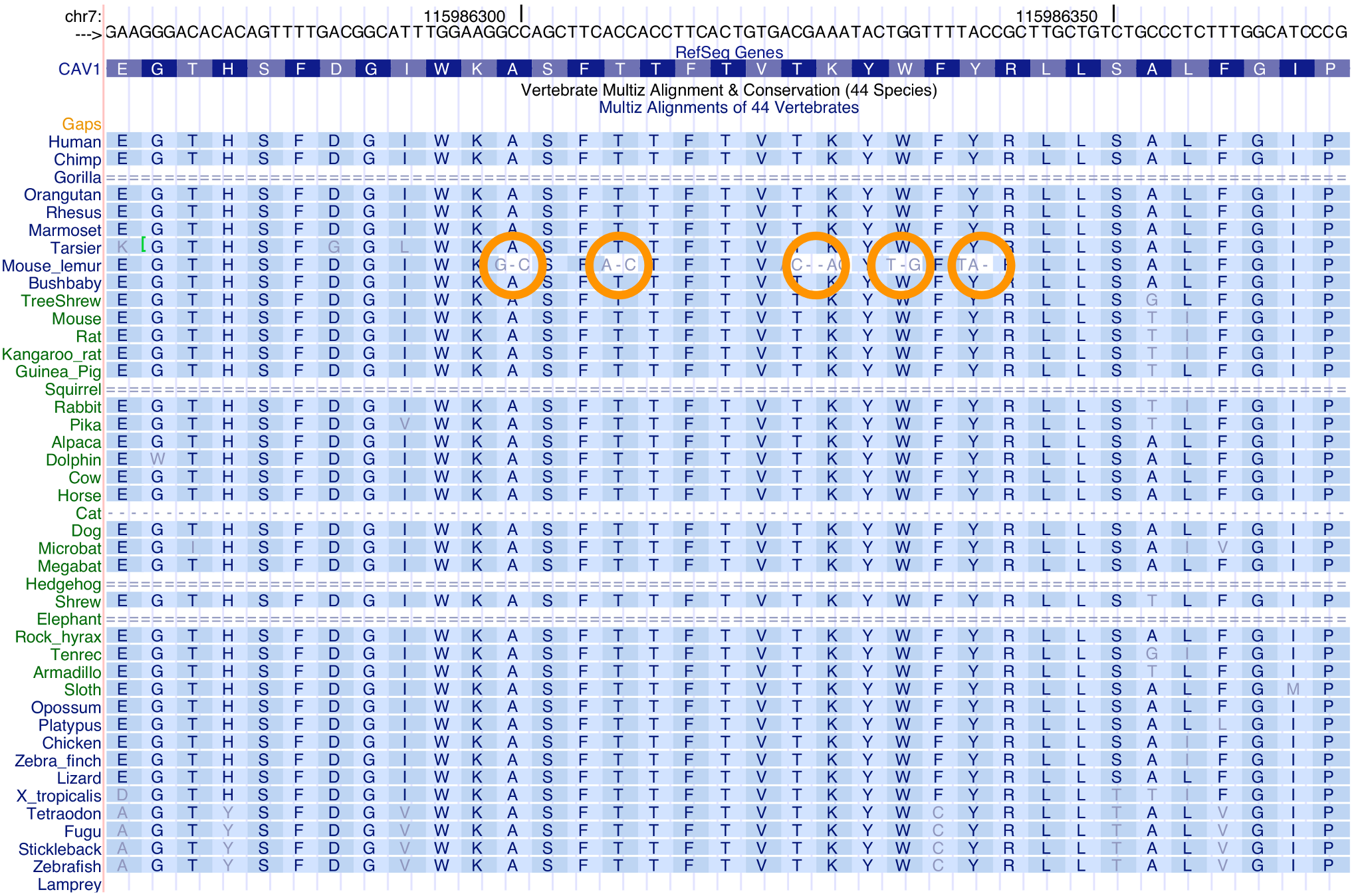

Supplement: Figure S2 — UCSC Genome Browser screen shot showing potential spurious indels in multiple alignment. Screen shot from the UCSC Genome Browser [6], showing the multiz-based genome-wide alignments—including the twenty low-coverage genome assemblies—for a coding exon of the caveolin I (CAV I) gene. Five apparent lineage-specific frame-shifting deletions in the mouse lemur sequence are shown. Comparative-grade sequence for mouse lemur from ENCODE region ENm001 indicates that these deletions reflect errors in the 2× assembly, rather than genuine deletion events in the evolution of the mouse lemur. This possibility is further supported by low phred scores in the 2× assembly (values of 3–23 flanking the deletions, and a mean score of 14 in the region), evidently reflecting single read coverage. Spurious indels of this kind can have numerous damaging consequences in phylogenomic analyses, for example, by distorting inferred indel rates, by causing comparative gene finders to fail, or by producing false signatures of accelerated lineage-specific evolution. While it is somewhat unusual to observe a tight cluster of five indels in one sequence, as in this case, spurious indels in coding regions are generally common in the 2× alignments (see text). (TIF) [file pone.0017034.s002.tif]

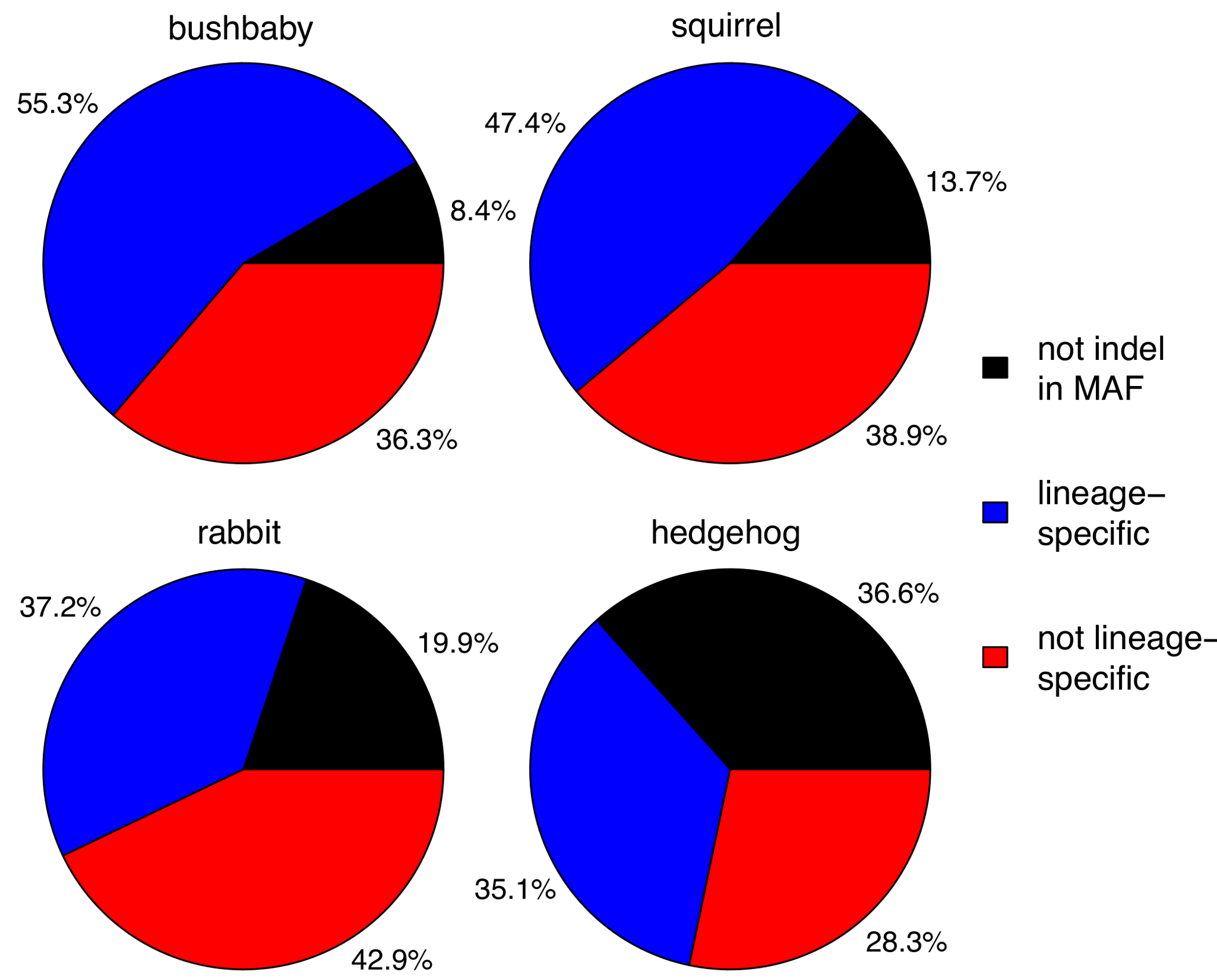

Supplement: Figure S3 — Fraction of indels in the 2×/ENCODE alignments which can be mapped to the 44-way alignment. The black slices represent the fraction of these indels in which no corresponding indel appears in the 44-way alignment, and may be explained by polymorphism. The blue slices appear as lineage-specific indels in the 44-way alignment, and are candidates for error mitigation when the quality score is sufficiently low. The red slices appear as non-lineage-specific indels and cannot be addressed by our method. These may be explained by alignment error or old polymorphism segregating in several species. (TIF) [file pone.0017034.s003.tif]

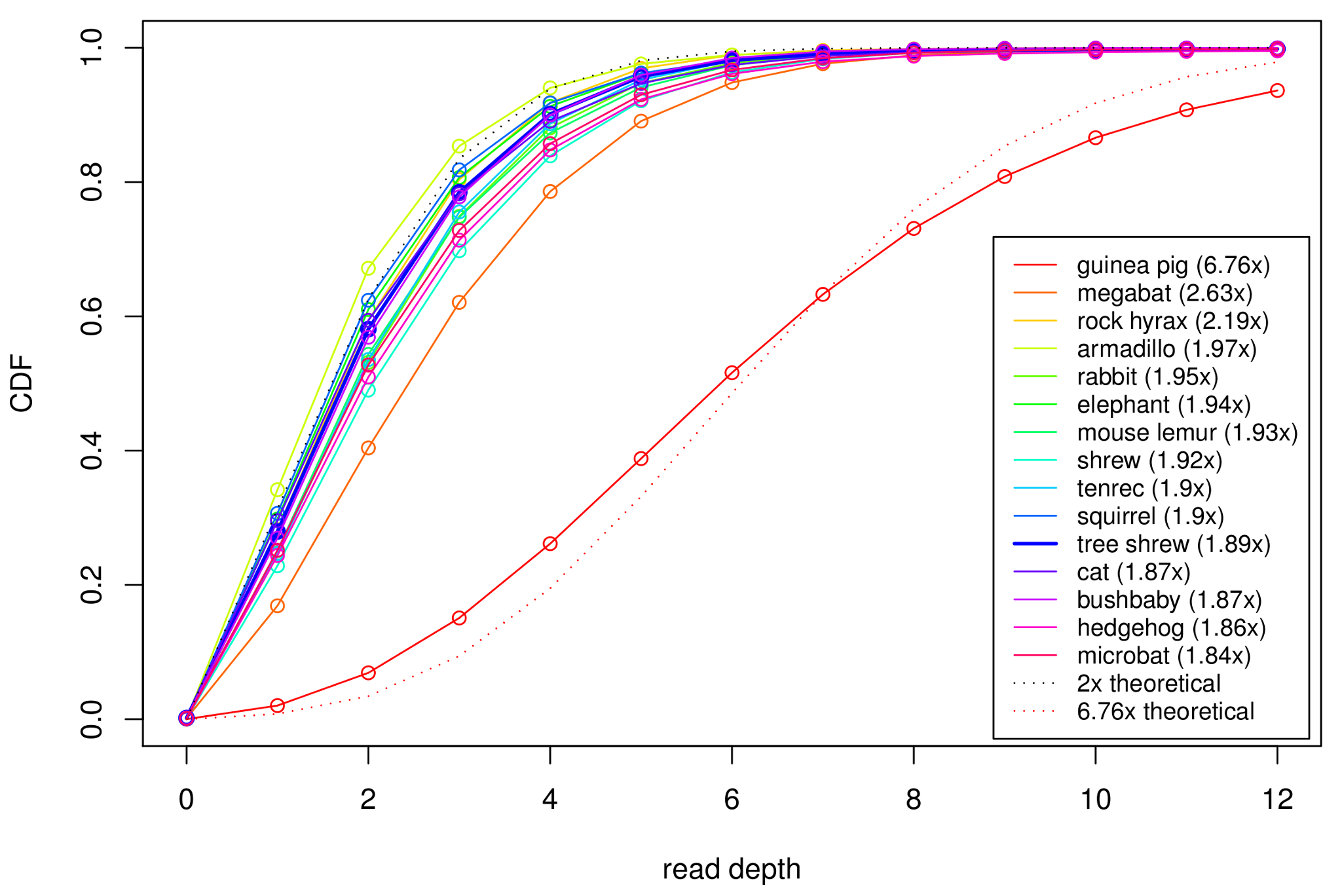

Supplement: Figure S4 — Empirical cumulative distribution functions (CDFs) for read depths in various assemblies. The distributions expected under comparable theoretical (Poisson) model are shown for comparison. Read depth was estimated from the assembly.reads file produced by the ARACHNE assembler. This file contains information about approximate placement of each read in the assembly. Because these placements are not exact, the depths should also be considered approximate. (TIF) [file pone.0017034.s004.tif]

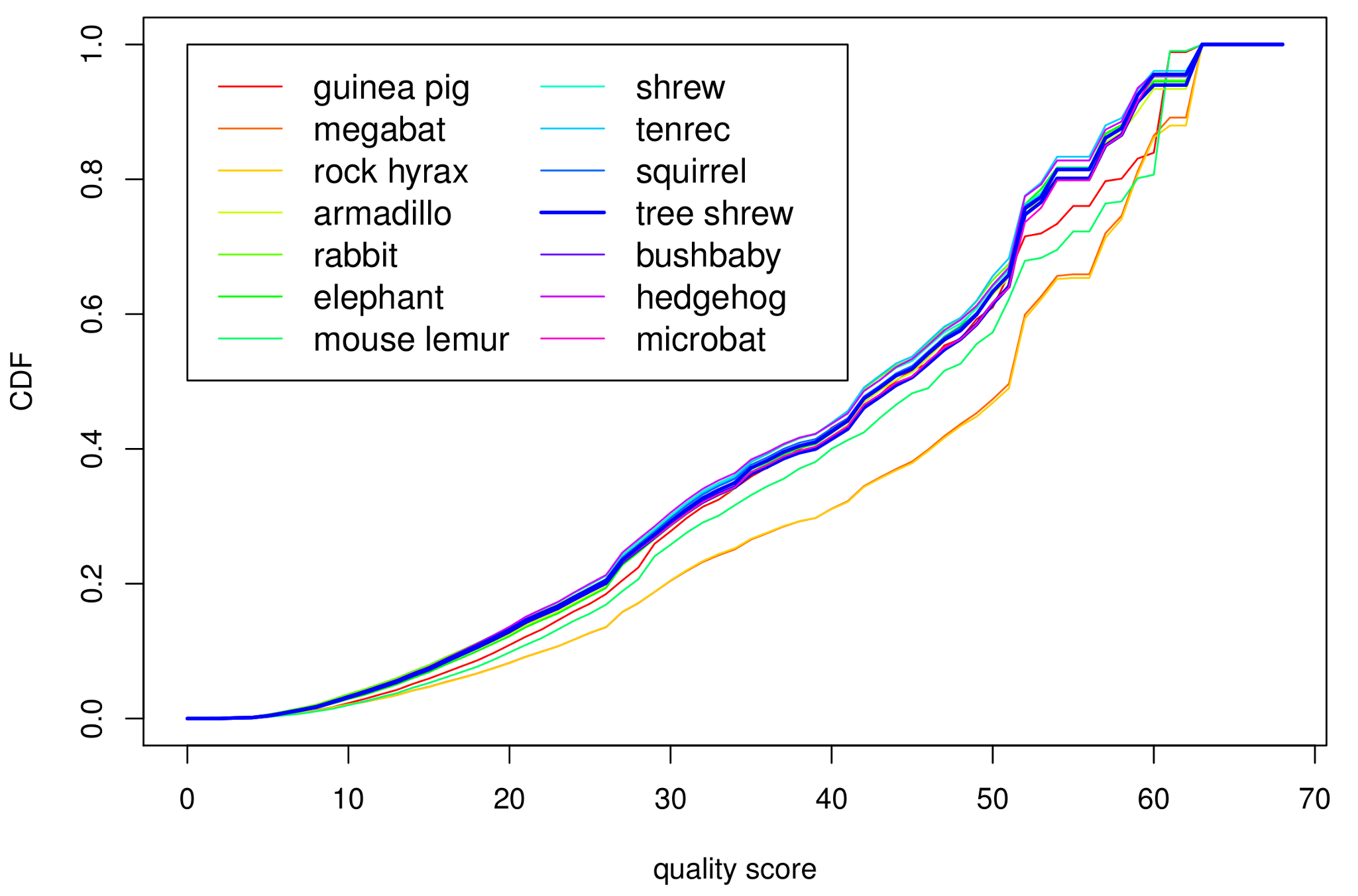

Supplement: Figure S5 — Empirical cumulative distribution functions (CDFs) for single-read quality scores in various assemblies. This figure is based on a random sample of ∼10,000 reads per assembly, and only represents the portion of each read which is used in the assembly. Tree shrew (shown in bold) was chosen as a representative species whose distribution was used in our model for coverage and error. (TIF) [file pone.0017034.s005.tif]

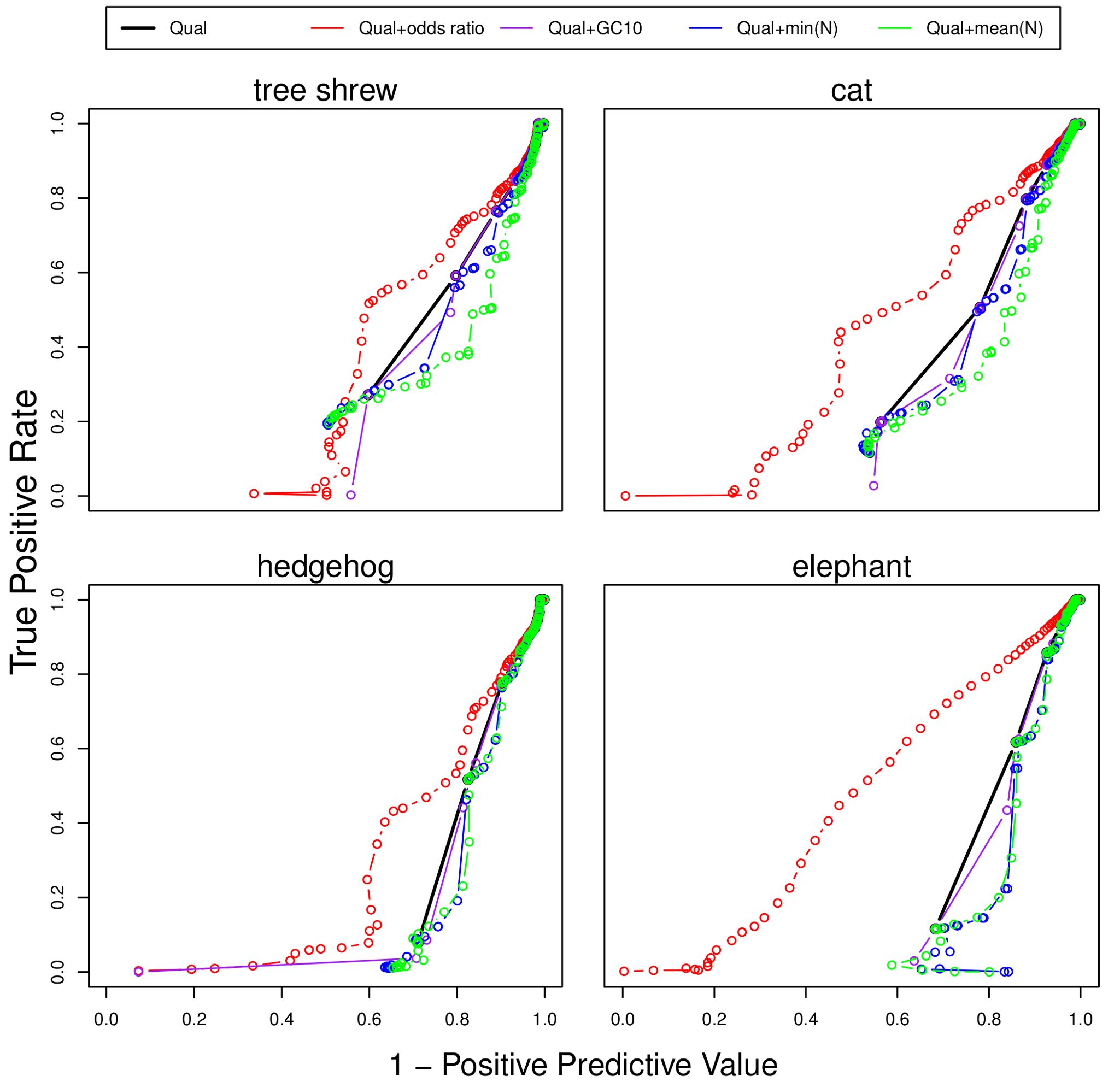

Supplement: Figure S6 — Performance of base masking algorithms which use linear-regression framework, for four species. The dark black lines show the baseline results using only quality scores. In red are the results where regression is performed on the quality scores and a phylogenetic odds ratio comparing a model with error to a model without error. In purple, blue, and green, respectively, are results which use both quality score and GC percentage in a 10 bp window, the minimum quality score in a 10 bp window, and the mean quality score in a 10 bp window. The only factor which seems to have a positive effect on the prediction is the phylogenetic odds ratio. (TIF) [file pone.0017034.s006.tif]
