# Supplementary material for: Error and Error Mitigation in Low-Coverage Genome Assemblies
Source: PLoS One. 2011 Feb 14;6(2):e17034. doi: 10.1371/journal.pone.0017034 (PMC3038916; doi:10.1371/journal.pone.0017034)
Supplement: Table S1 — Species nomenclature. (PDF) [file pone.0017034.s007.pdf]

# Error and Error Mitigation in Low-Coverage Genomes

**M.J. Hubisz, M.F. Lin, M. Kellis, A. Siepel**

Table S1: Species nomenclature

| Scientific name | Common name | ENCODE sequence name | 2x sequence name |
|---|---|---|---|
| *Dasypus novemcinctus* | Nine-banded Armadillo | armadillo | dasNov2 |
| *Echinops telfairi* | Lesser Hedgehog Tenrec | tenrec | echTel1 |
| *Erinaceus europaeus*[a] | European Hedgehog[a] | hedgehog | eriEur1 |
| *Felis catus* | Domestic Cat | cat | felCat3 |
| *Loxodonta africana* | African Savannah Elephant | elephant | loxAfr2 |
| *Microcebus murinus* | Gray Mouse Lemur | mouse_lemur | micMur1 |
| *Myotis lucifugus* | Little Brown Bat[b] | sbbat | myoLuc1 |
| *Oryctolagus cuniculus* | European Rabbit[c] | rabbit | oryCun1 |
| *Otolemur garnettii* | Northern Greater Galago[d] | galago | otoGar1 |
| *Procavia capensis* | Rock Hyrax | rock_hyrax | proCap1 |
| *Pteropus vampyrus* | Malayan Flying Fox[e] | flying_fox | pteVam1 |
| *Sorex araneus* | Common Shrew[f] | shrew | sorAra1 |
| *Spermophilus tridecemlineatus* | 13-Lined Ground Squirrel | st_squirrel | speTri1 |
| *Tupaia belangeri* | Northern Tree Shrew | tree_shrew | tupBel1 |
| *Cavia porcellus* | Guinea Pig | guinea_pig | cavPor3 |

[a]The Middle-African Hedgehog, *Atelerix albiventris*, was sequenced by ENCODE, whereas the European hedgehog *Erinaceus europaeus* was sequenced for the 2x project

[b]Also called Microbat

[c]The breed of the ENCODE rabbit is New Zealand White

[d]Also called Bushbaby

[e]Also called Megabat

[f]Listed as European Shrew by ENCODE project, and Common Shrew by the 2x project
