# Supplementary material for: Error and Error Mitigation in Low-Coverage Genome Assemblies
Source: PLoS One. 2011 Feb 14;6(2):e17034. doi: 10.1371/journal.pone.0017034 (PMC3038916; doi:10.1371/journal.pone.0017034)
Supplement: Table S2 — Summary of alignments of 2× genomes and corresponding ENCODE sequences. (PDF) [file pone.0017034.s008.pdf]

Error and Error Mitigation in Low-Coverage Genomes

M.J. Hubisz, M.F. Lin, M. Kellis, A. Siepel

Table S2: Summary of alignments of 2x genomes and corresponding ENCODE sequences

2x assembly	ENCODE species	ENCODE regions	Alignment length (Mb)	Percent coverage[a]
dasNov2	armadillo	44	13.79	38.3
echTel1	tenrec	42	11.15	37.7
eriEur1	hedgehog	39	10.97	39.1
felCat3	cat	43	12.69	46.5
loxAfr2	elephant	44	16.90	48.8
micMur1	mouse_lemur	43	19.18	64.4
myoLuc1	sbbat	41	13.99	59.7
oryCun1	rabbit	43	16.46	53.3
otoGar1	galago	44	20.05	53.7
proCap1	rock_hyrax	6	3.79	55.6
pteVam1	flying_fox	5	4.30	83.9
sorAra1	shrew	41	13.90	57.0
speTri1	st_squirrel	44	14.42	53.5
tupBel1	tree_shrew	4	2.14	53.5
cavPor3[b]	*guinea_pig*	*41*	*25.05*	*95.9*

[a]Fraction of ENCODE bases aligned to 2x assembly
[b]Sequenced to ∼7x coverage and shown for comparison.

Table S1. Estimates of $d_{\mathrm{N}}/d_{\mathrm{S}}$ for chr22 genes and four primates

**branch**	**full data**	**high-quality only**	**SEM**
tarsier	0.179	0.164	0.165
mouse lemur	0.173	0.166	0.162
bushbaby	0.189	0.179	0.171
tree shrew	0.135	0.123	0.124
internal branch	0.161	0.142	0.156

Table S2. CONGO performance with and without SEM

**Gene Set**	**Statistic**[a]	**Baseline (%)**[b]	**B+SEM (%)**[c]	**SEM (%)**[d]
CCDS[e]	Exon Sn	82.84	82.88	82.96
	Exon Sp	76.86	77.18	77.38
	Missed Exons	12.66	12.71	12.99
	Wrong Exons	17.01	16.95	16.86
	Nuc Sn	84.81	85.10	85.21
	Nuc Sp	82.13	82.08	82.02
R+E+U+G[f]	Exon Sn	74.26	74.31	74.36
	Exon Sp	89.88	90.28	90.49
	Missed Exons	21.86	21.90	22.17
	Wrong Exons	3.01	2.94	2.88
	Nuc Sn	76.01	76.30	76.40
	Nuc Sp	96.89	96.88	96.80

[a]As defined by [**?**]. Performance is measured against the whole genome, excluding the ENCODE "random" regions ($\sim$0.5% of the genome), which were used for training.

[b]Training and testing on original alignments.

[c]Training on original alignments, testing on alignments processed by SEM.

[d]Training and testing on alignments processed by SEM.

[e]"Consensus CDS" gene set (more conservative).

[f]Union of RefSeq, ENSEMBL, UCSC, and GENCODE gene sets (less conservative).

Table S3. CONGO performance by chromosome

	Exon Sp (%)[a]		Exon Sn (%)[b]	
**Chrom.**	**Baseline**	**SEM[c]**	**Baseline**	**SEM[c]**
1	90.10	**90.67**	83.00	**83.11**
2	90.34	**90.75**	85.52	85.43
3	90.43	**90.81**	86.27	86.05
4	89.43	**89.79**	84.75	**84.76**
5	89.33	**89.54**	85.75	85.69
6	89.16	**90.21**	84.05	**84.48**
7	90.61	**91.42**	81.99	**82.31**
8	89.53	**90.07**	85.05	**85.18**
9	89.97	**90.72**	84.53	**84.61**
10	90.02	**90.43**	83.94	83.66
11	86.51	**87.35**	84.52	**84.86**
12	91.11	**91.64**	85.83	85.83
13	90.62	**90.97**	81.85	**82.05**
14	88.55	**89.43**	86.19	**86.38**
15	91.28	**91.98**	83.00	**83.07**
16	89.99	**90.61**	79.69	**79.86**
17	90.93	**91.65**	83.53	**83.83**
18	89.24	**89.85**	88.07	**88.40**
19	89.93	**90.45**	71.70	**71.83**
20	91.38	**92.13**	85.24	**85.70**
21	91.36	**92.09**	81.76	**82.04**
22	90.83	**91.53**	80.33	**80.59**
X	88.06	**88.88**	74.80	**75.04**
Y	68.42	68.42	5.13	5.13

[a]Computed from union of RefSeq, ENSEMBL, UCSC, and GENCODE gene sets (R+E+U+G).
[b]Computed from CCDS gene set.
[c]SEM was used for training and testing. Bold indicates an improvement.
