# Supplementary material for: Error and Error Mitigation in Low-Coverage Genome Assemblies
Source: PLoS One. 2011 Feb 14;6(2):e17034. doi: 10.1371/journal.pone.0017034 (PMC3038916; doi:10.1371/journal.pone.0017034)
Supplement: Table S3 — Estimates of d N/ d S for chr22 genes and four primates. (PDF) [file pone.0017034.s009.pdf]

Error and Error Mitigation in Low-Coverage Genomes

M.J. Hubisz, M.F. Lin, M. Kellis, A. Siepel

Table S3: Estimates of $d_{\mathrm{N}}/d_{\mathrm{S}}$ for chr22 genes and four primates

**branch**	**full data**	**high-quality only**	**SEM**
tarsier	0.179	0.164	0.165
mouse lemur	0.173	0.166	0.162
bushbaby	0.189	0.179	0.171
tree shrew	0.135	0.123	0.124
internal branch	0.161	0.142	0.156
