# Supplementary material for: Error and Error Mitigation in Low-Coverage Genome Assemblies
Source: PLoS One. 2011 Feb 14;6(2):e17034. doi: 10.1371/journal.pone.0017034 (PMC3038916; doi:10.1371/journal.pone.0017034)
Supplement: Table S4 — CONGO performance with and without SEM. (PDF) [file pone.0017034.s010.pdf]

Error and Error Mitigation in Low-Coverage Genomes

M.J. Hubisz, M.F. Lin, M. Kellis, A. Siepel

Table S4: CONGO performance with and without SEM

**Gene Set**	**Statistic**[a]	**Baseline (%)**[b]	**B+SEM (%)**[c]	**SEM (%)**[d]
CCDS[e]	Exon Sn	82.84	82.88	82.96
	Exon Sp	76.86	77.18	77.38
	Missed Exons	12.66	12.71	12.99
	Wrong Exons	17.01	16.95	16.86
	Nuc Sn	84.81	85.10	85.21
	Nuc Sp	82.13	82.08	82.02
R+E+U+G[f]	Exon Sn	74.26	74.31	74.36
	Exon Sp	89.88	90.28	90.49
	Missed Exons	21.86	21.90	22.17
	Wrong Exons	3.01	2.94	2.88
	Nuc Sn	76.01	76.30	76.40
	Nuc Sp	96.89	96.88	96.80

[a]As defined by [1]. Performance is measured against the whole genome, excluding the ENCODE "random" regions (∼0.5% of the genome), which were used for training.

[b]Training and testing on original alignments.

[c]Training on original alignments, testing on alignments processed by SEM.

[d]Training and testing on alignments processed by SEM.

[e]"Consensus CDS" gene set (more conservative).

[f]Union of RefSeq, ENSEMBL, UCSC, and GENCODE gene sets (less conservative).

References

1. Burset M, Guigó R (1996) Evaluation of gene structure prediction programs. Genomics 34: 353-367.
