# Supplementary material for: Error and Error Mitigation in Low-Coverage Genome Assemblies
Source: PLoS One. 2011 Feb 14;6(2):e17034. doi: 10.1371/journal.pone.0017034 (PMC3038916; doi:10.1371/journal.pone.0017034)
Supplement: Table S5 — CONGO performance by chromosome. (PDF) [file pone.0017034.s011.pdf]

Error and Error Mitigation in Low-Coverage Genomes

M.J. Hubisz, M.F. Lin, M. Kellis, A. Siepel

Table S5: CONGO performance by chromosome

	**Exon Sp (%)[a]**		**Exon Sn (%)[b]**	
**Chrom.**	**Baseline**	**SEM[c]**	**Baseline**	**SEM[c]**
1	90.10	**90.67**	83.00	**83.11**
2	90.34	**90.75**	85.52	85.43
3	90.43	**90.81**	86.27	86.05
4	89.43	**89.79**	84.75	**84.76**
5	89.33	**89.54**	85.75	85.69
6	89.16	**90.21**	84.05	**84.48**
7	90.61	**91.42**	81.99	**82.31**
8	89.53	**90.07**	85.05	**85.18**
9	89.97	**90.72**	84.53	**84.61**
10	90.02	**90.43**	83.94	83.66
11	86.51	**87.35**	84.52	**84.86**
12	91.11	**91.64**	85.83	85.83
13	90.62	**90.97**	81.85	**82.05**
14	88.55	**89.43**	86.19	**86.38**
15	91.28	**91.98**	83.00	**83.07**
16	89.99	**90.61**	79.69	**79.86**
17	90.93	**91.65**	83.53	**83.83**
18	89.24	**89.85**	88.07	**88.40**
19	89.93	**90.45**	71.70	**71.83**
20	91.38	**92.13**	85.24	**85.70**
21	91.36	**92.09**	81.76	**82.04**
22	90.83	**91.53**	80.33	**80.59**
X	88.06	**88.88**	74.80	**75.04**
Y	68.42	68.42	5.13	5.13

[a]Computed from union of RefSeq, ENSEMBL, UCSC, and GENCODE gene sets (R+E+U+G).

[b]Computed from CCDS gene set.

[c]SEM was used for training and testing. Bold indicates an improvement.
