# Supplementary material for: Error and Error Mitigation in Low-Coverage Genome Assemblies
Source: PLoS One. 2011 Feb 14;6(2):e17034. doi: 10.1371/journal.pone.0017034 (PMC3038916; doi:10.1371/journal.pone.0017034)
Supplement: Methods S1 — Estimation of indel rates. (PDF) [file pone.0017034.s012.pdf]

Error and Error Mitigation in Low-Coverage Genomes

M.J. Hubisz, M.F. Lin, M. Kellis, A. Siepel

Supplemental Methods S1: Estimation of indel rates

We estimated relative rates of insertion/deletion events on each branch of the tree using a maximum likelihood approach. As described in the Methods section (under "Indel imputation"), we identify indel regions (IRs) and represent them as matrices of 0s and 1s in a collapsed form, under the assumption that the probability of an insertion or deletion does not depend on its length. As in the parsimony case, we consider a $2^n \times 2^n$ transition matrix, but in this case we assume that transitions occur according to a continuous-time Markov process with infinitesimal generator $\mathbf{Q}^{(n)} = \{q_{ij}^{(n)}\}$ $(1 \leq i, j \leq 2^n)$ such that

$$q_{ij}^{(n)} \propto \begin{cases} 1 & \text{if } i \rightarrow j \text{ requires a single insertion} \\ \beta & \text{if } i \rightarrow j \text{ requires a single deletion} \\ -\sum_{k \neq i} q_{ik}^{(n)} & \text{if } i = j \\ 0 & \text{otherwise} \end{cases} \tag{1}$$

The matrix is normalized so that the expected rate at stationarity equals n, i.e., such that $\sum_{i,j:i \neq j} \pi_i^{(n)} q_{ij}^{(n)} = n$ where $\boldsymbol{\pi}^{(n)} = (\pi_1^{(n)}, \ldots, \pi_{2^n}^{(n)})$ is the stationary distribution (obtained by eigendecomposition). This ensures that branch length units can be interpreted as expected numbers of events per position.

We used an expectation-maximization algorithm, similar to one described previously [1], to estimate the values of β and the branch lengths $\mathbf{t} = \{t_v : \text{branches } v\}$. For the expectation (E) step, we used the sum-product algorithm to compute the joint posterior probability of the starting and ending states at each branch, given the states at the leaf nodes and the current parameter estimates. These probabilities are summed across indel regions to quantities of the form $c_{vuw}^{(n)}$, representing the expected count of transitions from each parent state u to each child state w on each branch v for a particular indel length n. This algorithm also yields posterior probabilities for each state at the root node, which are summed to compute $c_{Ru}^{(n)}$, the expected fraction of sites having state u at the root for a given value of n. Then, the expected complete log likelihood, for observed data X and unobserved data Y (the ancestral

indel states), is given by:

$$E[\log P(X, Y|\mathbf{t}, \beta)] = \sum_{n} \left(\sum_{u} c_{Ru}^{(n)} \log \pi_{u}^{(n)} + \sum_{v} \sum_{u} \sum_{w} c_{vuw}^{(n)} \log p(u|w, t_v) \right). \quad (2)$$

Here, $p(u|w, t_v)$ is determined in the usual way by exponentiating $\mathbf{Q}$, i.e., $p(u|w, t_v) = [\exp(\mathbf{Q}t_v)]_{w,u}$. For the maximization (M) step, we adjust β and the branch lengths $\mathbf{t}$ to maximize this quantity, numerically using the BFGS algorithm. The E and M steps were repeated until the parameters converged. Because of the computational complexity of the estimation, we allowed for a maximum value of $n = 3$ for the results shown in Figure 5, which discards the most complicated 10.1% of the indel regions.

The indel rate estimation was performed on multiple alignments for chromosome 22. It was repeated for the raw data, an error-mitigated data set using a quality threshold $q < 25$, and a third high-quality data set in which any species with a quality score $q < 45$ within a particular region was treated as missing data. Figure 5 was then created by computing the percent difference in branch lengths between both of the first two data sets and the high quality set. The medians and quartiles are taken from the distribution of these values within each group of branches.

References

1. Siepel A, Haussler D (2004) Phylogenetic estimation of context-dependent substitution rates by maximum likelihood. Mol Biol Evol 21: 468-488.
