# Supplementary material for: Error and Error Mitigation in Low-Coverage Genome Assemblies
Source: PLoS One. 2011 Feb 14;6(2):e17034. doi: 10.1371/journal.pone.0017034 (PMC3038916; doi:10.1371/journal.pone.0017034)
Supplement: Methods S2 — Estimation of d N/ d S. (PDF) [file pone.0017034.s013.pdf]

Error and Error Mitigation in Low-Coverage Genomes

M.J. Hubisz, M.F. Lin, M. Kellis, A. Siepel

Supplemental Methods S2: Estimation of $d_{\mathbf{N}}/d_{\mathbf{S}}$

We measured the nonsynonymous to synonymous substitution rate ratio ($d_{\mathrm{N}}/d_{\mathrm{S}}$) under two different models, using our own software implementation [1]. Three versions of aligned coding regions from chromosome 22 were used: one containing the original data, a second masked with threshold $q < 20$, and a third masked with threshold $q < 45$ and therefore containing only very high-quality sequence. Because of the computational complexity of phylogenetic likelihood calculations on large data sets, we used a subset of the species, consisting of tree shrew, bushbaby, mouse lemur, and tarsier.

In the first model we estimated a different value of $\omega = d_{\mathrm{N}}/d_{\mathrm{S}}$ for each branch of the tree, as well as a scaling factor for the entire tree (μ). The relative branch lengths were given by the neutral tree as computed using fourfold degenerate sites. As shown in Table S3, the full (raw) data set produced estimates of ω roughly 5–10% higher than those based on the high-quality data set only, evidently due to the effects of sequencing error. Once the masking (SEM) procedure was applied, the estimates of ω were reduced essentially to the levels observed with high-quality data—except for the case of the internal branch, which may be affected more by alignment error than by sequencing error. These estimates are based on very large data sets and the observed differences, while small, are almost certainly statistically significant (although we have not done a formal test).

The second model was a site model similar to the one introduced by [2]. In this case, the branches share common values of ω, but there are two categories of sites. The neutral category is constrained to have $\omega_{\mathrm{N}} \leq 1$, and the selected category has $\omega_{\mathrm{Sel}} > 1$. Each site comes from the selected category with probability p_{Sel}, and otherwise it is neutral. We estimated p_{Sel}, ω_{Sel}, ω_{N}, and the scaling factor μ. We focus in our comparison on the parameter p_{Sel} as an indication of the overall evidence for positive selection. The full data set led to an estimate of $p_{\mathrm{Sel}} = 0.131$, compared with $p_{\mathrm{Sel}} = 0.102$ for the high-quality data set, suggesting that sequencing error tends can inflate evidence for positive selection. After applying the SEM procedure, the estimate decreased substantially, to $p_{\mathrm{Sel}} = 0.119$.

References

1. Kosiol C, Vinar T, da Fonseca R, Hubisz M, Bustamante C, et al. (2008) Patterns of positive selection in six mammalian genomes. PLoS Genet 4: e1000144.

2. Nielsen R, Yang Z (1998) Likelihood models for detecting positively selected amino acid sites and applications to the HIV-1 envelope gene. Genetics 148: 929-936.
