# Supplementary material for: Error and Error Mitigation in Low-Coverage Genome Assemblies
Source: PLoS One. 2011 Feb 14;6(2):e17034. doi: 10.1371/journal.pone.0017034 (PMC3038916; doi:10.1371/journal.pone.0017034)
Supplement: Methods S3 — CONGO exon-finding analysis. (PDF) [file pone.0017034.s014.pdf]

Error and Error Mitigation in Low-Coverage Genomes

M.J. Hubisz, M.F. Lin, M. Kellis, A. Siepel

Supplemental Methods S3: CONGO exon-finding analysis

To assess how error correction can improve methods for functional element identification, we tested the CONGO comparative protein-coding exon predictor (Lin & Kellis, in prep.) using both our original alignments and a version that had been processed by automatic sequencing error mitigation (SEM). Like other modern methods for comparative gene prediction (for review, see [1]), CONGO uses a statistical machine learning algorithm to combine several lines of evidence, including codon substitution frequencies, indel and frameshift patterns, and splice site signal detectors, but without using any experimental evidence such as transcript sequences.

Measuring the standard "Exon Sn" and "Exon Sp" performance statistics, which require exact agreement between predictions and annotations [2], the error-corrected alignments led to a significantly improved specificity without detracting from sensitivity (Table S4). This improvement was observed when we applied the CONGO model trained on the original alignments to SEM-processed alignments, and further enhanced when we both trained it on and applied it to the SEM-processed alignments (with no other methodological changes). For example, the original Exon Sp measured against pooled annotations of the major human gene catalogs (excluding the regions used to train CONGO's graphical model) was 89.88%, which improved to 90.28% with the original model applied to the error-corrected alignments, and further improved to 90.49% when the model was retrained for the error-corrected alignments. Meanwhile, exact recovery of exons in the high-quality CCDS set increased slightly from 82.84% to 82.96%. The improvement in specificity held across individual chromosomes, while the sensitivity was slightly variable (Table S5).

Overall, the improvement from the error correction was significant but not dramatic, amounting to a $\sim$6% reduction in the false positive rate measured in this way (some of the "false positives" could represent still-unknown coding exons). Also, the results were mixed when we measured performance by allowing any amount of overlap between predictions and annotations (see "Missed Exons" and "Wrong Exons" in Table S4), suggesting that the error correction was more helpful for identifying the correct boundaries of coding exons than determining their presence or absence.

To a certain extent, probabilistic models such as semi-Markov conditional random fields, which underlie CONGO, inherently tolerate noise in the input data, by quantitatively weighing adverse observations (e.g. frameshifts

or stop codons) based on the frequency of such events in training data, rather than immediately disqualifying a prediction based on fixed rules. Thus, it is not very surprising that the error correction yielded to only a modest improvement in its performance. Notably, the relative weight that CONGO's machine learning procedure assigned to frameshifting indels increased by about 20% in the retrained model, suggesting that observed frameshifts are indeed more reliable in the error-corrected alignments. We conclude that the significant improvement in exact exon prediction performance indicates that the error correction increases the reliability of the 2X sequences, immediately leading to improved results even for advanced, already noise-tolerant probabilistic methods.

References

1. Brent MR (2008) Steady progress and recent breakthroughs in the accuracy of automated genome annotation. Nat Rev Genet 9: 62–73.

2. Burset M, Guigó R (1996) Evaluation of gene structure prediction programs. Genomics 34: 353-367.
