# Supplementary material for: Error and Error Mitigation in Low-Coverage Genome Assemblies
Source: PLoS One. 2011 Feb 14;6(2):e17034. doi: 10.1371/journal.pone.0017034 (PMC3038916; doi:10.1371/journal.pone.0017034)
Supplement: Methods S4 — Theoretical model. (PDF) [file pone.0017034.s015.pdf]

Error and Error Mitigation in Low-Coverage Genomes

M.J. Hubisz, M.F. Lin, M. Kellis, A. Siepel

Supplemental Methods S4: Theoretical model

Here we provide a more detailed description of the theoretical model that is sketched in the main paper. We first assume that the read depth at each base is Poisson-distributed, with mean λ equal to the average coverage (e.g., $\lambda = 2$ for a 2x assembly). Therefore, given that a base has been sequenced, its read-coverage x is distributed as

$$p(x|x > 0; \lambda) = \frac{\text{Pois}(x; \lambda)}{1 - \text{Pois}(x = 0; \lambda)} = \frac{\lambda^x e^{-\lambda}}{x!(1 - e^{-\lambda})}. \tag{1}$$

This assumption appears to hold fairly well in practice for our data, although most assemblies do show a slight depletion of low-coverage bases and enrichment of high-coverage bases (Figure S4), perhaps due to cloning biases. Second, we assume that true error rates are well predicted by nominal quality scores, as suggested by our empirical data (Figure 2). Third, we assume independence of quality scores across reads, so that with a read depth of x at some position i, the joint distribution of quality scores at i, $p(q_{i,1}, q_{i,2}, \ldots, q_{i,x})$, is equal to a product of marginal distributions, $p_1(q_{i,1})p_1(q_{i,2}) \cdots p_1(q_{i,x})$, where $p_1(q)$ is the probability of observing a quality score q in an individual read. Notice that this property does not require that quality scores are identically distributed along each read (which is clearly not true; see Figure S1), as long as the offsets of overlapping reads are uniformly distributed. Finally, we assume that a quality score for an assembled base can be accurately expressed as a sum of the quality scores in the individual reads.

These assumptions are not strictly consistent with empirical observations, but we expect them to hold approximately for real data. Furthermore, they allow several quantities of interest to be computed easily using only the distribution of quality scores for single reads, $p_1(q)$, which, as noted, can be estimated empirically. By the read-independence and score-additivitity assumptions above, the distribution of quality scores for any specific depth-of-coverage x, $p_x(q)$, is given by an x-wise convolution of p_1, which can be computed recursively, for modestly large x, using the relation

$$p_x(q) = \sum_{q'} p_1(q')p_{x-1}(q - q'). \tag{2}$$

The overall distribution of quality scores for an assembly with average coverage λ can then be computed by

marginalizing with respect to the Poisson-distributed read depths:

$$p(q|\lambda) = \sum_{x=1}^{\infty} \frac{\lambda^x e^{-\lambda}}{x!(1-e^{-\lambda})} p_x(q). \tag{3}$$

This quantity can be approximated arbitrarily closely by evaluating the sum up to a sufficiently large x_{max}. We set x_{max} to the 0.999 quantile of the Poisson distribution with mean λ.

Assuming that quality scores accurately predict error rates, the expected overall error rate corresponding to this distribution is given by

$$\text{err}(\lambda) = \sum_q p(q|\lambda) 10^{-q/10}. \tag{4}$$

For convenience of interpretation, we express this quantity in *phred* units and denote it Q^*, with

$$Q^*(\lambda) = -10\log_{10}(\text{err}(\lambda)) = -10\log_{10}\left(\sum_q p(q|\lambda)10^{-q/10}\right). \tag{5}$$

It is worth noting that Q^* is a generalized f-mean of the basewise quality scores, with $f(q) = 10^{-q/10}$. Similarly, the error rate in regions of x-fold coverage, expressed in *phred* units, is

$$Q_x = -10\log_{10}\left(\sum_q p_x(q)10^{-q/10}\right). \tag{6}$$

The fraction of the total error that comes from regions of single coverage is therefore

$$F_1 = \frac{\lambda^1 e^{-\lambda}}{1!(1-e^{-\lambda})} \cdot \frac{10^{-Q_1/10}}{10^{-Q^*(\lambda)/10}} = \frac{\lambda e^{-\lambda}}{1-e^{-\lambda}} \cdot 10^{(Q^*(\lambda)-Q_x)/10}. \tag{7}$$

As shown in Figure 3, our estimates of p_1 imply that $Q^*(\lambda)$ is closely approximated by a measure of error that considers only single-coverage regions of the assembly, which we denote $\tilde{Q}^*$. This quantity has a simple closed-form expression, and can be used to approximate Q^* for any λ:

$$\tilde{Q}^* = -10\log_{10}\left(\sum_q \frac{\lambda e^{-\lambda}}{1-e^{-\lambda}} p_1(q)10^{-q/10}\right) = Q_1 - 10\log_{10}\left(\frac{\lambda e^{-\lambda}}{1-e^{-\lambda}}\right) \tag{8}$$

The last term above is fairly well approximated by the linear function 3.5λ in the range of interest. Based on our data, $Q_1 = 19.7$. Thus, to a first approximation, $\tilde{Q}^* \approx 20 + 3.5\lambda$.
